# Novel Genomes of *Sphingomonadales* Strains Isolated from Diverse Environments

**DOI:** 10.3390/microorganisms14030698

**Published:** 2026-03-20

**Authors:** Nathan W. Williams, Tahir Ali, Paul D. Boudreau

**Affiliations:** Department of BioMolecular Sciences, University of Mississippi School of Pharmacy, Oxford, MS 38677, USA

**Keywords:** soil microbiome, biofilms, cyanobacterial microbiome, whole-genome sequencing

## Abstract

Glycosphingolipids are amphiphilic compounds that feature sugar or glycan moieties installed onto a ceramide lipid. The synthesis of glycosphingolipids by members of the human gut microbiome, and their known immune stimulating activity, have made them of interest for potential pharmaceutical roles. However, the known diversity of glycosphingolipid glycans in bacteria remains limited, highlighting the need to isolate novel glycosphingolipid-producing organisms as a source of these compounds. The order *Sphingomonadales*, one of the major clades of sphingolipid producing bacteria, conserves a serine palmitoyltransferase (SPT) enzyme needed for the initial biosynthetic step in sphingolipid production which can be targeted as part of isolation efforts. With these bacteria known to live in diverse environments such as soil microbiomes, soap scum biofilms, and cyanobacterial microbiomes, there are many environments to target for the isolation of these bacteria. In this work, we designed a set of polymerase chain reaction (PCR) primers for the isolation of diverse *Sphingomonadales* strains by targeting the SPT gene (*spt*), which we used to isolate strains from the genera *Sphingomonas* and *Novosphingobium* in soil, soap scum biofilms, and xenic cyanobacterial cultures. In these efforts, streptomycin improved the encounter rate, as represented by the SPT assay true-positive rate. Our isolates represent novel genomic space: with genomes from both genera that have low similarity to known genomes, suggestive of novel species, while several novel plasmids were also missing known marker sequences.

## 1. Introduction

Inspired by the observation that the glycosyltransferase responsible for installing glucuronic acid on ceramide characterized in *Zymomonas mobilis* has homologs widely distributed across the order *Sphingomonadales* [1,2], this work centers on the isolation of novel bacteria within this clade to build a strain library to study for novel glycosphingolipid chemistry. Glycosphingolipids, such as α-galactosylceramide (α-Gal), are amphiphilic compounds containing a ceramide lipid backbone and a sugar/glycan moiety that are known to be CD1d-based agonists for stimulating the immune system [3,4]. Bacterial lipids are commonly used as vaccine adjuvants, though glycolipids can have toxicity concerns, and work with monophosphoryl lipid A has been promising [5,6]. Glycosphingolipids, such as α-Gal, have yet to be successfully applied in this role, but clinical trials have explored this application [7,8,9,10]. The immune-stimulating role of bacterial sphingolipids is also seen natively in the human gut microbiome, where *Bacteroides*-derived sphingolipid deficiency is correlated with chronic inflammation of the bowel [11,12]. Work elucidating bacterial glycosphingolipid biosynthesis, which was poorly understood compared to the eukaryotic pathway, has made great strides recently, though several glycosyltransferases involved in the glycan biosynthesis remain unknown [1,2,13,14]. In this way, there is growing interest in understanding the diversity and biosynthesis of bacterial glycosphingolipid glycans.

A first step in these efforts is to isolate novel sphingolipid-producing bacteria. For this purpose, a polymerase chain reaction (PCR)-based screening protocol for the serine palmitoyltransferase (SPT) gene (*spt*), the first biosynthesis step in the production of sphingolipids, was developed [13,15]. Early work testing enrichment media led to the isolation of two novel species of *Novosphingobium* [2]. Since then, these primers led to the isolation of a third strain of *Novosphingobium* [16]. To further expand our *Sphingomonadales* bacterial library, we searched through the literature to identify accessible environments known to be colonized by members of the order *Sphingomonadales* that could be targeted for isolation efforts.

This literature search first led to the many reports that *Sphingomonadales* bacteria have been isolated from the soil, including representatives of genera such as *Sphingomonas*, *Novosphingobium*, *Sphingobium*, and *Sphingopyxis* [17,18,19,20,21,22,23]. These versatile genera have adapted to different environments ranging across the rhizosphere of plants, contaminated soil, wetlands, sediment, volcanic rock, and farm soil [19,20,21,23,24,25]. With these bacteria being isolated from polluted environments, it is unsurprising that these genera are being examined for their bioremediation and aromatic compound-degrading properties [19,20,21,23,24,25,26]. Furthermore, some of these soil isolates have even shown interesting microbiological activities, such as antibiotic degrading capabilities [21]. Collectively, these works suggested the soil microbiome was a good environment to target for the isolation of these bacteria.

An article which revealed α-proteobacteria, represented by the genus *Sphingomonas*, can be abundant among soap scum biofilms, suggested that this environment might also be worth exploring [27]. Biofilms of these bacteria not only provide protection against desiccation, but contain proteins that are corrosive, and can degrade copper pipes [28,29]. Water systems have been found to be contaminated by biofilms containing *Sphingomonas*, which may be because they are adaptable, e.g., having the ability to utilize multiple chain length carbon sources [30]. Given this, the built environments such as potable water systems and soap scum residues was suspected to house species from the genus *Sphingomonas* [18,27].

Apart from this literature survey, preliminary sequencing work on strains of cyanobacteria (collected as part of a separate research project), detected the presence of *Sphingomonadales* bacteria as members of the heterotrophic microbiomes of cyanobacteria in the Boudreau laboratory’s culture collection [31]. Prompted by this observation, a targeted inspection of the literature on cyanobacterial microbiomes revealed reports of *Sphingopyxis*, *Sphingobium*, and *Zymomonas* species from strain isolation and 16S rRNA gene sequencing efforts [32,33,34,35]. *Sphingobium* was found to dominate the heterotrophic microbiome of cyanobacteria under certain light conditions, where this genus may play a key role in nitrogen nutrient cycling [35]. Furthermore, *Sphingomonadales* bacteria have shown degrading capabilities of cyanobacterial heptapeptide hepatotoxins [35,36]. Which suggested these strains may have important ecological roles in cyanobacterial microbiomes, making xenic cyanobacterial culture collections an interesting source from which to isolate *Sphingomonadales* bacteria.

In this report we detail our isolation, and whole genome sequencing of, novel *Sphingomonadales* strains from the soil, anthropogenic environments (e.g., soap scum biofilms), and xenic cyanobacterial cultures. Insights garnered from this work can inform and improve future isolation efforts.

## 2. Materials and Methods

For details of the media preparation and other general laboratory parameters see the Methods Supplement in SI File S1.

### 2.1. Environmental Sampling and Bacterial Isolation

#### 2.1.1. Soil Samples

Soil samples were collected along the walking trail from The University of Mississippi Museum to the Rowan Oak Museum and other locations on the University of Mississippi campus. Subsamples of 1 g were then weighed out from the soil samples and suspended in 1 mL of sterile phosphate buffered saline (PBS) with 1 h of gentle rocking. After which, dilutions of 5× and 50× were prepared and, unfiltered, 10 μL aliquots of the dilutions were spread on 1/5× Luria Broth (LB) plates and Defined Medium for Siderophores with Citric Acid (DMS-CA) plates (both treated with nystatin) before incubation at 27 °C. Plates were monitored for 21 days for bacterial colonies. Isolated distinct colonies from these plates were picked based on morphology using a sterile loop to transfer and spread on new plates of the same medium (but without nystatin) and incubated at 27 °C. Bacteria from restruck plates displaying a single morphology were tested for the presence of *spt* by PCR (see below). This was done for *Sphingomonas aurantiaca* BL-S-01 and strains *Novosphingobium* sp. BL-S-19 through *Novosphingobium* sp. BL-S-34. SPT-positive strains by this assay were then grown in 5 mL liquid culture of 1/5× LB for strains from 1/5× LB plates, or modified *Acidovorax* Complex Medium (see Methods Supplement in SI File S1) for strains grown on DMS-CA. The 5 mL liquid cultures were incubated until turbid and used for preparation of a 1:1 culture to sterile 50% glycerol (aq) frozen stock. Frozen stocks were vortexed briefly, then frozen at −70 °C for long-term storage.

#### 2.1.2. Soap Scum Biofilms

Biofilm samples were collected using sterilized bacterial swabs. Swabs were placed into 1 mL of PBS and allowed to incubate for 1 h with gentle rocking, serially diluted with PBS to 1/50× of the original concentration, and then spread with 10 µL on 1/5× LB plates and DMS-CA plates containing nystatin. Plates incubated at 27 °C and monitored over 14 days for colony formation. Colonies were picked based on distinct morphology and restruck on fresh plates and grown under the same conditions, until observation of a single morphology. This was done only for isolates *Sphingomonas hankookensis* BL-S-02 through *Sphingomonas* sp. BL-S-05. Isolates *Sphingomonas* sp. BL-S-06 through *Sphingomonas* sp. BL-S-18 were picked from plates of LB + STR and DMS-CA + STR agar lacking nystatin started directly by the swab, incubated at 25 °C, and monitored over 21 days. All pure colonies were tested by PCR (see below), grown in liquid culture, and preserved as frozen stocks as with the soil isolates (see Section 2.1.1). A 10% DMSO frozen stock was also made of the same liquid culture, vortexed briefly, and frozen at −70 °C for long-term storage.

#### 2.1.3. Cyanobacterial Heterotrophic Microbiomes

Cyanobacterial microbiome samples were collected from our laboratory’s continuous cultures of our cyanobacteria which are grown at 50 mL scale in BG-11. *Pseudanabaena* cyanobacterial strains BL-A-26, BL-A-28, and BL-A-41 were sampled [37]. A sterile 1 µL loop was dipped in the cyanobacterial culture and then struck directly on DMS-CA plates with nystatin and streptomycin. 10 and 50 µL of each liquid culture was also spread directly on separate plates. All plates were incubated at 25 °C and monitored for growth over 21 days. Individual distinct colonies were picked and restruck onto fresh plates, prepared with only streptomycin, based on morphology. A later second isolation attempt from strains BL-A-28 and BL-A-41 was run with 1/5× LB + STR plates struck out with 1 µL loops, colonies from which were restruck on 1/5× LB to achieve pure cultures. Pure colonies were PCR tested, grown in 1/5× LB liquid culture, and preserved as frozen stocks as with the soap scum biofilm isolates (see Section 2.1.2).

#### 2.1.4. SPT PCR Assay

The original PCR primers LT11/LT13 [15] were mapped to the *spt* homolog of *Novosphingobium capsulatum* B-4261 (BCYV01000023) in Geneious Prime (version 2024.0.7, with java version 11.0.20.1 + 1, 64 bit) and the corresponding sequence in B-4261 was used to design the primers PB05/PB06. The primers PB07/PB08 were designed via the same process with the *spt* homolog in *Zymomonas mobilis* subsp. *mobilis* B-14023 (CP006818).

The PCR was prepared with ice-cooled components, adding 1.6 μL each of the *spt* forward and reverse primer mixes (see SI Table S1), 40 μL of Taq 2× PCR master mix with dye (AB Clonal; Woburn, MA, USA), and 30.8 μL of sterile deionized water. This material was mixed by pipetting and distributed in equal volumes across a PCR sample eight-tube strip. 0.75 μL of DNA extract from a prior *Novosphingobium* sp. isolate was added to the first tube as a positive control [2,16]. For the rest of the tubes, 0.75 μL of MilliQ-purified water was used to pick a single colony from the isolation plate using a pipette, and the single colony suspension was added to a sample tube with the PCR mixture before being mixed by pipette. The PCR sample strip was spun down briefly, then loaded into the thermocycler to be immediately run with the *spt* PCR method (see SI Table S2). After completion of the PCR, the samples were then examined using gel electrophoresis. A 1.5% agarose (Sigma Life Sciences; St. Louis, MO, USA; for molecular biology EEO) gel was prepared in 1× TAE buffer with ethidium bromide (Sigma Aldrich; St. Louis, MO, USA; 10 mg/mL in H_2_O). New England Biolabs’ Quick-Loading Purple 1 kb Plus DNA Ladder was used as the length reference for PCR products. SPT-positive strains would result in an amplified band like the positive reference at ca. 1 kb, bands were judged loosely, bands could be slightly above or below the positive control, to not miss diverse sphingolipid-producing strains.

### 2.2. DNA Isolation and Sequencing

#### 2.2.1. Low-Molecular-Weight Genomic DNA for 16S rRNA Gene Sequencing

DNA was isolated from 3 mL of turbid culture (from the remaining volume grown for the cryogenic stock preparation, Section 2.1). First, 1.5 mL were centrifuged at 4000× *g* for 10 min at 14 °C and to the cell pellet, an additional 1.5 mL of culture added. With the second supernatant removed by repeating the centrifugation. DNA was isolated from the pellet based upon the manufacturer’s protocol for Omega Bio-Tek’s (Norcross, GA, USA) E.Z.N.A. Bacterial DNA kit. For this protocol, a heat block was used in replacement of a hot water bath, and samples were briefly vortexed every 20 min over a 1 h incubation. Columns were eluted twice; for BL-S-01 through BL-S-05 an initial volume of 75 µL of the vendor’s elution buffer warmed to 65 °C was incubated on the column at room temperature for 5 min followed by a second elution of 50 µL incubated at room temperature for 3 min. Samples BL-S-06 through BL-S-34 had the protocol modified so that the first volume was incubated on the column for 2.5 min at 65 °C followed by a 2.5 min incubation at room temperature, total incubation of the first volume was 5 min. The final 125 µL DNA extracts were quantified by fluorescence using the Qubit. Samples with a concentration ≤ 20.0 ng/μL were concentrated with a Zymo (Irvine, CA, USA) DNA Clean & Concentrator 5 kit. Following the manufacturer’s protocol, samples were eluted with 12 µL and then 8 µL elution buffer with centrifugation > 10,000× *g* at 13 °C, for 20 µL total when combined. All samples were prepared at a final minimum concentration range of 20–25 ng/μL, 10 µL of which was sent for 16S rRNA gene sequencing using the commercial vendor AZENTA Life Sciences (South Plainfield, NJ, USA), via their Bacterial Identification Service targeting the V1 through V9 regions.

#### 2.2.2. High-Molecular-Weight Genomic DNA Preparation

Cryogenic stocks were struck on plates of their original isolation enrichment medium and grown under the same conditions used to isolate the sample. Isolated colonies were used to inoculate 5 mL of liquid media by sterile loop. This liquid culture was grown to turbidity and then centrifuged with the same conditions as for 16S rRNA gene sequencing using only 3 mL of the culture. Pellets were left in the freezer overnight for use or up to a week later. For *Sphingomonas zeae* BL-S-08, *Sphingomonas zeae* BL-S-12, and the cyanobacterial microbiome heterotroph isolates, larger scale cultures were grown. A 5 mL liquid culture, as described above, was used as a starter culture to inoculate a larger liquid culture. Starter cultures were used when their measured absorbance at 660 nm was 0.5–0.8. Then, 45 µL of starter culture was used to inoculate 45 mL of media, which was grown to the same absorbance range prior to pelleting. The whole 45 mL cultures were centrifuged at 4000× *g* for 10 min. Pellets were subjected to high-molecular-weight (HMW) DNA extraction with the Macherey-Nagel (Allentown, PA, USA) Bio NucleoBond kit based upon the enzymatic lysis protocol for bacteria cells with modifications details as follows. Shaking was set for 140 RPM, mixed by inversion, centrifuged with 9000× *g* at 4 °C, and buffers were added dropwise. Pellets were transferred and re-weighed in a 50 mL tube. Exactly 10 µL of lysozyme (OMEGA Bio-Tek) was supplemented to the 1 mL of H1 buffer per 20 mg of wet cells suggested by the manufacturer’s protocol. Samples were then vortexed and incubated at 37 °C for 1 h. Next, H1 buffer was used to bring the total volume to 5 mL and 200 µL of proteinase K (Macherey-Nagel) was added followed by a 30 min incubation at 50 °C. An amount of 100 µL RNAase (Macherey-Nagel) was then added to the sample, mixed by inversion, and allowed to incubate at room temperature for 5 min. The sample was then column purified according to the manufacturer’s protocol, with HMW DNA eluted by H5 buffer into a new 50 mL falcon tube. The HMW DNA was precipitated with 2-propanol (Fisher Scientific; Atlanta, GA, USA) by inverting the tube 10 times and centrifuging for 10 min. Fresh 70% EtOH (diluted from Decon Laboratories; King of Prussia, PA, USA, 200 proof ethanol with MilliQ-purified water) was used to wash the pellet and was removed after centrifugation for 5 min. The pellet was air dried for 20–90 min to remove residual 70% EtOH and resuspended in HE (elution) buffer. Samples were incubated overnight on an orbital shaker. (Specific modifications to the protocol were: BL-S-02 and *Novosphingobium* sp. BL-A-28-Hi1-0D for which incubation speed was greater than 140 RPM, and with BL-S-02 the filter was washed with a stream instead of dropwise.) All sample concentrations were measured by fluorescence, the DNA concentrations, in ng/µL, were: 94.6 (BL-S-01), 42.9 (BL-S-02), 84.9 (BL-S-05), 56.0 (BL-S-08), 42.2 (BL-S-12), 59.0 (BL-S-19), 130.0 (*Novosphingobium* sp. BL-S-20), 63.0 (*Novosphingobium* sp. BL-S-21), 58.0 (*Novosphingobium* sp. BL-S-22), 55.0 (*Novosphingobium* sp. BL-S-23), 109.0 (*Novosphingobium* sp. BL-S-24) 251.0 (*Sphingomonas* sp. BL-S-25), 60.0 (*Sphingomonas bisphenolicum* BL-S-26), 365.0 (*Sphingomonas* sp. BL-S-27), 54.0 (*Sphingomonas* sp. BL-S-28), 171.0 (*Sphingomonas* sp. BL-S-29), 59.0 (*Sphingomonas* sp. BL-S-30), 58.0 (*Sphingomonas* sp. BL-S-31), 60.0 (*Novosphingobium* sp. BL-S-32), 59.0 (*Novosphingobium* sp. BL-S-33), 100.2 (*Novosphingobium* sp. BL-S-34), 19.6 (BL-A-28-Hi1-0D), 122.0 (*Novosphingobium* sp. BL-A-41-Hi4A-0G), and 53.0 (*Novosphingobium* sp. BL-A-41-Hi4B-X5A). The DNA extracts of BL-S-02, BL-S-12, and BL-A-28-Hi1-0D were subjected to bead-based size selection and concentration before sequencing (see below).

#### 2.2.3. Bead-Based Size Selection

Bead-based size selection was done at a 1:2 *v*/*v* ratio of bead suspension to sample to improve the DNA sample quality and obtain the vendor’s requested concentration (50 ng/µL). The HMW DNA sample and the calculated SERAMAG volume were added to a 1.5 mL microcentrifuge tube, which was then flicked by hand for 60 s, spun down briefly via a bench top centrifuge, and afterwards was allowed to incubate standing at room temperature for 7 min. The beads were pelleted on a magnetic rack for 5 min, then the supernatant was removed and discarded, while still on the magnetic rack. Beads were washed with 200 µL of 85% ethanol (made fresh from 200 proof ethanol, Decon Laboratories, diluted with MilliQ-purified water) added to the sides of the microcentrifuge tube above the pellet, to allow the solution to run over the pellet with as little disturbance as possible. The wash was removed and discarded without disturbing the pellet and the process repeated for a second wash. The beads were air dried for 7 min at room temperature. Afterwards, the microcentrifuge tube was removed from the magnetic rack to add 50 µL of TE buffer (see Section 2.2.1). The pellet was resuspended by flicking for 60 s, briefly spun down, and incubated standing at room temperature for 7 min, then for 5 min back on the magnetic rack. The supernatant was carefully transferred with a wide-bore pipette tip to a new microcentrifuge tube. Final DNA concentrations were measured by Qubit: 66.3, 57.7, and 48.3 ng/µL for BL-S-02, BL-S-12, and BL-A-28-Hi1-0D, respectively, which was deemed sufficient for whole-genome sequencing.

#### 2.2.4. Whole-Genome Sequencing

The HMW DNA samples were shipped to the vendor (Plasmidsaurus; Louisville, KY, USA) for nanopore sequencing (via their Standard Bacterial Genome sequencing service). The vendor used V14 library prep chemistry for R10.4.1 cells on a PromethION (Oxford Nanopore; Oxford, UK) with Dorado basecalling on super-accuracy mode to produce the raw genomic reads processed below.

### 2.3. Bioinformatics Analysis

#### 2.3.1. 16S rRNA Gene Sequencing Analysis

All .ab1 files received from AZENTA Life Sciences were processed in Geneious Prime. Each isolate had a pair of forward and reverse Sanger reads provided by the vendor’s 16S rRNA gene sequencing service. To process these files, the reverse reads were reverse complimented and then all .ab1 files were subjected to an automated trim for both the 5’ and 3’ ends at an Error Probability Limit of 0.005. The Geneious prime Align/Assemble tool was used to pairwise align the forward and reverse reads of each isolate, with alignment settings of a global alignment with free end gaps, cost matrix: 65% similarity (5.0/−4.0), gap open penalty of 12, and a gap extension penalty of 3. Alignments with more than one ambiguity in the sequence were trimmed further, manually. To manually trim, the sequence was inspected outward from the consensus region where both reads overlapped. Ambiguous bases were trimmed such that a maximum of 1 ambiguity to the left, and 1 ambiguity to the right, of the overlapped region remained. All the consensus sequences, post manual and/or automated trimming, within the same putative genus were compared against each other with the Geneious prime alignment tool. Multiple alignments were set up using Geneious alignment tool with the parameters as above. These alignments showed multiple isolates that were not distinct based on 16S rRNA gene sequence (see SI Table S3). To save on cost, a representative of each sequence type was selected for HMW DNA extraction rather than whole-genome sequencing all of them.

#### 2.3.2. Genome Assembly

Raw .fastq files from Plasmidsaurus were processed on a System 76 computer running Pop!_OS 22.04 with a 6.0 GHz Core i9-14900K (24 Core/32 Thread) processor, 32 GB of DDR5 4800 MHz (1 × 32) memory, a 1 TB PCIe4 M.2 solid state drive, and an NVIDIA 8 GB GeForce RTX 4060 Ti graphics card. The raw .fastq files were filtered using the Filtlong tool (version v0.2.1) [38], first with settings for a minimum length of 500 bp and then by quality score to keep only the top 99% of reads (with no filtering by quality if read removal by length cutoff already removed 1% or more of reads). Then, a second filtered file was prepared from the same raw .fastq files using Filtlong with cutoffs at a minimum length of 1000 bp and the top 90% of reads by quality. The 500 bp/99% filtered reads were assembled via Flye (version 2.9.5-b1801) with the nanopore high quality reads setting [39]. Flye results revealed each assembly had multiple contigs with good coverage of the putative chromosomes (minimum 37× with BL-S-34, see SI Table S4). The initial Flye assembly was polished with the 1000 bp/90% reads using medaka (version 2.0.1) with the r1041_e82_400bps_bacterial_methylation configuration file and a batch size set to 32 [40]. The result of this was the medaka-polished consensus .fasta file. Based on assessment of the Flye-derived assembly graph (the presence of short, less than 50 kb, linear contigs), or circular contigs with unreasonably short lengths (less than 10 kb), the assemblies were trimmed manually of short low-quality contigs. Specifically, contig 3 from BL-S-02, contigs 1, 3, 10, 12 and 14 from BL-S-12, contig 4 from BL-S-22, and contigs 2 and 3 from BL-S-29 were manually removed. After this trimming, the final versions were made available on GenBank.

#### 2.3.3. Type Strain Genome Server (TYGS) Analysis

All whole genome sequenced medaka polished files were uploaded to the TYGS website [41]. No restriction was applied, though genomes assessed as similar were run together in a batch. For more information on this analysis see the Methods Supplement in SI File S1.

An important note is that while the initial 16S gene sequence of the strain BL-S-28 was *Sphingomonas*-like, the later genome-derived 16S gene was clearly *Novosphingobium*-like, suggestive of contamination of the culture used to prepare the high-molecular-weight DNA (SI Figure S4), as such the dubious genome sequence of BL-S-28 was excluded from later analysis.

#### 2.3.4. Assignment of Assembled Contigs as Chromosomal, Plasmid, or Viral Sequences

The final genome assemblies were examined for potential phage contamination and the presence of plasmids with geNomad (version v1.11.0, using geNomad with the end-to-end and cleanup workflow commands). This analysis did not suggest any phage contamination of the genomes, and convincingly scored several contigs as plasmids (see SI Tables S4–S6) [42]. To further validate this analysis, medaka-polished assembly files were also submitted to DFAST annotation (version 1.3.6, using the web platform). In Geneious prime, the DFAST annotations were examined for the putative plasmids that were not scored by geNomad, these contigs were searched for plasmid and virus associated genes; however, no hits were found. These unscored plasmids were also compared to plasmids from *Sphingomonas* or *Novosphingobium* strains available on GenBank via the OrthoANI Tool (version 0.93.1) (see below) [43]. Separately, the DFAST annotations of the plasmid-scored megabase-length contigs were interrogated for ribosomal RNA genes and when found the 16S rRNA genes were aligned in Geneious against the 16S rRNA genes of that strain’s larger chromosome to ensure they had identity and did not represent contamination of the pure culture. These assignments of plasmid versus chromosomal DNA were included in the description of the contigs published on GenBank.

#### 2.3.5. OrthoANI Tool (OAT) Comparison

OAT (version 0.93.1) was used to compare ortho average nucleotide identity (orthoANI) of the genomes against the nearest clades from the TYGS phylogenetic tree [41,43]. OAT settings were set to use 1 to 4 threads, calculating the orthoANI in both directions with averaging used to reconcile different values, and a Genome-to-Genome Distance Calculator setting of 2. The four plasmids which were not scored by the geNomad analysis were extracted as single contigs in Geneious and run through the same analysis against other plasmids from GenBank, as well as scored plasmids from this work, using the same OAT settings as with the genomes.

A final large-scale analysis was run on the Linux computer, using the cmd.jar version of the OAT with version 2.16.0+ of the BLAST database to compare the strains from this work against all available reference genomes on GenBank within the “Sphingomonadaceae (Sphingomonas group)” taxa at the chromosome or complete level of assembly (as of 15 October 2025).

## 3. Results

### 3.1. Novel Sphingomonadales Strains Were Isolated from Diverse Environments

#### 3.1.1. Isolation from Soil Microbiomes

The first isolate, BL-S-01, was obtained from a soil sample collected from the University of Mississippi campus walking trail between Rowan Oak and the University of Mississippi Museum. While strains BL-S-19 through BL-S-34 were obtained from soil samples collected from different locations across the University of Mississippi campus. Notably, BL-S-01 was isolated without streptomycin selection, in contrast to the isolations of BL-S-19 through BL-S-34. A preliminary identification by 16S rRNA gene sequencing confirmed these isolates as members of the order *Sphingomonadales*, with a nearest hit, by pairwise identity, to strains from the genera *Sphingomonas* or *Novosphingobium* (SI Table S3). This preliminary sequencing also showed several strains with identical 16S rRNA genes. Strains BL-S-01 and BL-S-19 through BL-S-34 were selected for whole-genome sequencing analysis.

#### 3.1.2. Isolation from the Anthropogenic Environment–Soap Scum Biofilms

Isolates BL-S-02 through BL-S-18 were collected from soap scum biofilms taken from residential bathrooms. BL-S-02 through BL-S-05 were isolated on DMS-CA, while BL-S-06 through BL-S-18 were isolated on either LB + STR or DMS-CA + STR. Preliminary identification by 16S rRNA gene sequencing confirmed isolates as part of the *Sphingomonadales* with the nearest hits, via pairwise identity, in the genus *Sphingomonas* (SI Table S3). A total of 17 SPT-positive isolates were identified from soap scum biofilms. To narrow down candidate strains for whole-genome sequencing, we prioritized strains to ensure each unique 16S rRNA gene was represented by a whole genome sequence. Strains BL-S-02, BL-S-05, BL-S-08, and BL-S-12 were selected for whole-genome sequencing analysis.

#### 3.1.3. Isolation from Cyanobacterial Heterotrophic Microbiomes

Heterotrophic isolates from the Boudreau Lab’s xenic cyanobacteria and algae culture collection were collected from two cultures *Pseudanabaena* sp. BL-A-28 and *Pseudanabaena* sp. BL-A-41. The initial batch of isolates was isolated on DMS-CA + STR, while the second batch of isolates was collected on 1/5× LB + STR. Preliminary identification by 16S rRNA gene sequencing confirmed that all the isolates were *Sphingomonadales* with the nearest hit, by pairwise identity, to the genus *Novosphingobium* (SI Table S3), as expected from the prior metagenomic sequencing of the BL-A-41 cyanobacterial culture [31]. 16S rRNA gene sequencing suggested that all the initial heterotrophic isolates from BL-A-28 were identical to each other, with the same being true of the isolates from BL-A-41. So, one isolate from each algal source was selected for whole-genome sequencing. In the second batch of isolates, one BL-A-41 isolate was distinct from all other BL-A-41 isolates, so that strain was whole-genome sequenced too. These strains were named according to a different coding system to reflect their origin as isolates from a cyanobacterial culture, first the cyanobacterial strain number is given (e.g., BL-A-28), then “Hi” to represent a heterotrophic isolate with an identifier for each species found in the microbiome, and a strain identifier for each distinct isolated colony. Strains BL-A-28-Hi1-0D, BL-A-41-Hi4A-0G, and BL-A-41-Hi4B-X5A were selected for whole-genome sequencing.

#### 3.1.4. Streptomycin Resistance and Colony Pigmentation Were Useful for Identifying *Sphingomonadales* Strains

Comparing the initial isolation efforts from the soil and soap scum biofilms, it was clear that the false-positive rate of the PCR assay was lower when streptomycin was used to select for *Sphingomonadales* strains. In the initial soil isolation efforts without streptomycin selection, only 1 of 20 SPT-positive isolates was a true-positive hit. While in later isolations using streptomycin selection and prioritizing the yellow pigmentation of *Sphingomonadales* colonies, the encounter rate increased. With the soap scum biofilms, initial efforts without streptomycin showed eight SPT-positive isolates, of which four were true hits representing two unique isolates (by 16S sequence). Culturing from the biofilms with streptomycin, 13 SPT-positive isolates were obtained, all of which were true hits representing three unique isolates (by 16S sequence). Showing a stark increase in true positive rate with streptomycin use. A caveat with this analysis is that false-positive PCR profiles were recorded and used to exclude hits in later PCR runs, so the PCR screen became more discerning over time. However, the finding that streptomycin, resistance to which is common across the *Sphingomonadales*, is a good selection method for *Sphingomonadales* isolation agrees with the prior literature [17,18].

### 3.2. Whole Genome Sequences of the Sphingomonadales Isolates Showed Novel Genomic Space

#### 3.2.1. Whole Genome Sequence Assemblies Were Complete and Had Numerous Plasmids

After nanopore sequencing of the high-molecular-weight DNA extractions with the vendor (Plasmidsaurus), the raw sequence data was processed, assembled, and initial assemblies were polished and curated to afford the final genomes. Genomes of these isolates were assembled at 4.09 to 6.04 Mb (Table 1), with closed circular chromosomes (except for BL-S-31), with numerous plasmids observed (SI Table S4). Several of the genome assemblies had two megabase-length contigs; in all cases, the shorter of these was scored as a plasmid by the geNomad annotation pipeline [31]. Except for contig 1 in BL-S-19, each of these potential megabase-length plasmids was annotated by the DFAST pipeline as bearing rRNA genes. The presence of these central housekeeping genes led us to annotate these megabase-length contigs as secondary chromosomes rather than plasmids. An alignment of the rRNA genes between the two chromosomes confirmed their identity weighing against the possibility of contamination of the culture with a second *Sphingomonadales* strain.

#### 3.2.2. Whole Genome-Based Phylogenies Identified Potentially Novel Species

**Figure 1:**
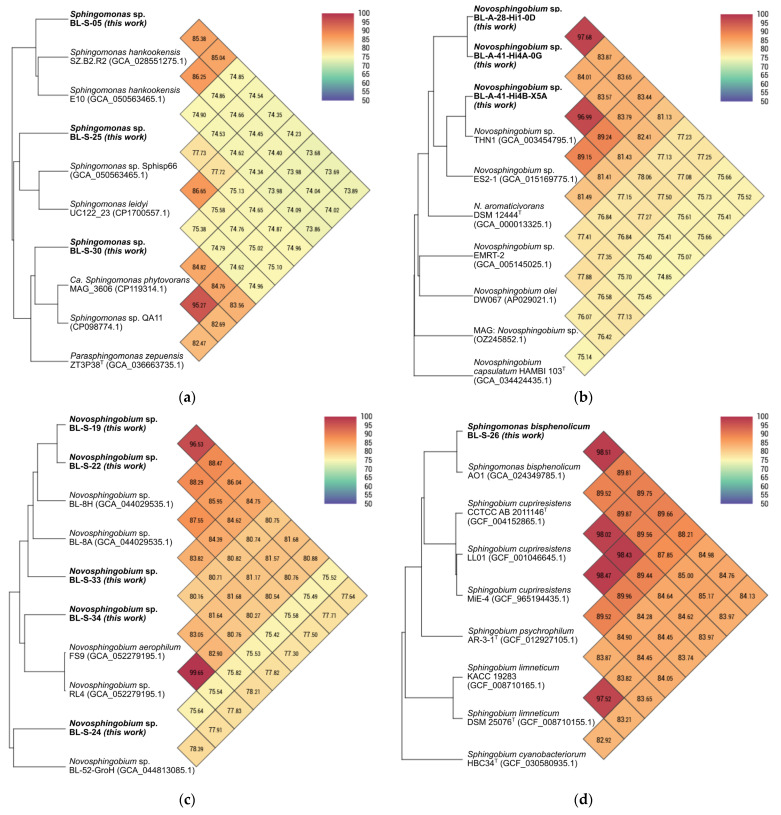
OrthoANI comparison of representative genomes against top hits across the genera *Novosphingobium*, *Parasphingomonas*, *Sphingobium*, or *Sphingomonas*. Representative genomes were selected of the: (**a**) *Sphingomonas* isolates; (**b**) *Novosphingobium* isolates from cyanobacterial cultures; (**c**) the soil *Novosphingobium* isolates; (**d**) and *Sphingomonas bisphenolicum* BL-S-26 which appears to clade with the genus *Sphingobium* not *Sphingomonas*. Heatmaps were generated with the OAT [43].

A phylogenetic comparison of the final assemblies was made via the Type Strain Genomic Server (TYGS), which showed our strains clading with the genera *Novosphingobium*, *Parasphingomonas*, *Sphingobium*, or *Sphingomonas* (SI Figures S1–S9) [33]. This analysis supported the assignment that many of the isolates were novel species. To further this analysis, the OrthoANI Tool (OAT) was used to compare our genomes to some of their top hits from the TYGS analysis and across the genera (Figure 1) [41,43]. Here comparison against genomes on GenBank with complete/chromosome level assemblies was prioritized. This enhanced comparison supported most of our isolates as being candidate novel species with poor orthoANI scores to known genomes (e.g., Figure 1a). This analysis also allowed the assignment of BL-S-01 as a member of the species *S. aurantiaca* (SI Figure S10a), BL-S-02 as a *S. hankookensis* (SI Figure S10b), and BL-S-08 and BL-S-12 as both being *S. zeae* (SI Figure S10c). Both the TYGS and OAT analysis supported BL-S-26 as being within the species *S. bisphenolicum*; however, that species is not validly published on the list of prokaryotic names with standing in nomenclature [44]. Indeed, while our analysis showed BL-S-26 and the nomenclatural type strain (AO1) are highly similar, the TYGS and OAT analysis claded both within the *Sphingobium* not the *Sphingomonas* (Figure 1d). Further chemotaxonomic analysis will be needed to properly assess whether BL-S-26 and AO1 are better described as *Sphingobium* or *Sphingomonas*; we use the nomenclature *Sphingomonas bisphenolicum* for BL-S-26 as it is highly similar to AO1 and we have yet to carry out such an assessment. Similarly, the strains BL-S-27, BL-S-29, BL-S-30, and BL-S-31 claded with both representatives of the genera *Sphingomonas* and the closely related *Parasphingomonas* (Figure 1a), a more in-depth chemotaxonomic analysis will be needed to assess whether these strains should be revised to the genus *Parasphingomonas*.

Close (>96%) orthoANI scores could not be found when comparing the other genomes to known species available on GenBank. An OAT-based comparison against all *Sphingomonadaceae* assemblies on GenBank with complete- or chromosome-level assemblies supported the initial TYGS assignment of these genomes as new species (SI File S2), though a more thorough chemotaxonomic assessment would be necessary to validate that assessment. Notably, one comparison greater than 96% was found between *Novosphingobium* sp. BL-A-41-Hi4B-X5A and *Novosphingobium* sp. THN1 (GCA_003454795.1), but the latter strain lacks a species description (Figure 1b).

#### 3.2.3. Bioinformatic Analyses Showed the Isolated Strains Contain Novel Plasmids

The assemblies contained several small (<1 Mb) circular contigs that we initially presumed were plasmids, however the analysis with geNomad did not score four of them as either potential phage genomes or plasmids (see SI Table S4). However, using the OAT to compare these four contigs against other *Sphingomonadales* plasmids, all showed high (>90%) hits to other known plasmids from this clade of bacteria (SI Figure S11a–c), supporting their assignment as novel *Sphingomonadales* plasmids [43].

## 4. Discussion

### 4.1. Successful Isolation and Sequencing of Novel Sphingomonadales Strains

As we used profiles of PCR bands from initial false positive strains to exclude later screened colonies, determining the exact false positive rate for this workflow is difficult. Without streptomycin, 88 picked colonies yielded 28 positive hits, only five of which were true positives; with streptomycin, 71 picked colonies yielded 44 positive hits, of which 34 were true positives. Giving an absolute true positive rate of 25% without and 77% with streptomycin selection.

DMS-CA was originally designed for the isolation of siderophores [45]; however, the isolation of BL-S-01, BL-S-05, BL-A-Hi1-0D, and BL-A-41-Hi4A-0G on this medium is in line with the prior isolation of *Novosphingobium* isolates utilizing this medium [2,16]. This work suggests that this medium is a good choice for the isolation of novel *Sphingomonadales* bacteria. Future work will be needed to assess our strains’ abilities to resist heavy metals or produce siderophores as this is not widely explored in this genus, though members of the genus *Novosphingobium* have been studied for hydrocarbon degradation purposes [19,46].

Our investigation across diverse environments, including the isolation of several potentially novel species, supports the resistance to streptomycin being widespread in the genera *Novosphingobium* and *Sphingomonas* [2,17,18,47,48]. With each isolate reported here displaying the yellow colony pigmentation that is characteristic of the order *Sphingomonadales*, this work also supports the screening of colonies by pigmentation for the targeted isolation of this clade.

### 4.2. Implications for Microbial Ecology and Strain Library Utility

The *Novosphingobium* spp. isolates were seen to clade distinctly between the cyanobacterial microbiome and soil microbiome isolates (Figure 1b,c). While soil *Novosphingobium* isolates had low similarity to known genomes, the most similar genomes were prior Boudreau lab isolates collected from the same region. It is perhaps not surprising that the *Novosphingobium* would clade based on their environmental origin as adaptation to the environment is likely to be a key driver of differentiation within the genus. Though this result does strongly support the benefit of sampling from different environmental sources and that future *Sphingomonadales* isolation efforts should target multiple environments.

A key result, and benefit for the larger field, of this work is the deposition of twenty-three novel genomes publicly on GenBank. In future work, our lab will compare the glycosyltransferase genes present in this strain library to guide the discovery of novel glycosphingolipid biosynthetic enzymes. Similarly, other labs will be able to explore other potential genes of interest and metabolic pathways in these genomes.

### 4.3. Method Limitations

While the PCR primers used in this study successfully guided the isolation based on the presence of the *spt* gene [15], the false-positive rate was higher than ideal. Initial efforts may have been overly tolerant in accepting bands as positive even when they were above or below the standard amplicon’s length. Since the goal was to be able to screen microbes from diverse environments (the soil, soap scum biofilms, and cyanobacterial and algal microbiomes), high fidelity of the primer pairs to the target gene is essential. In future work, we will compare the use of other primer pairs to reduce the false positive rate in screening for the *spt* gene or the use of novel primers that target other highly conserved domains such as pigment biosynthesis genes.

One of the limitations of this work is not having metagenomic sequencing of the original soil samples (which we omitted to control costs). As such, it is not possible to assess if there were *Sphingomonadales* strains abundant in the soil which were missed in this workflow because they could not be cultured on the chosen media or could not be amplified by the PCR primers. With the cost of sequencing decreasing, future work would benefit from using raw soil metagenomic sequencing to better understand the bias of this workflow as well as direct sequencing of the *spt* amplicons to help validate the proper targeting of this gene.

## Figures and Tables

**Table 1:** Basic genomic characteristics of the whole-genome sequenced strains.

Strain	BL-S-01	BL-S-02	BL-S-05	BL-S-08	BL-S-12	BL-S-19	BL-S-20
Genome Size (Mb)	4.19	4.25	4.34	4.49	4.85	5.95	5.13
G + C Content (%)	66.2	66.7	66.1	65.6	65.4	64.1	64.7
Completeness/ Contamination *	99.19%/ 1.62%	98.82%/ 1.39%	98.59%/ 2.73%	99.57%/ 2.05%	99.86%/ 3.35%	97.15%/ 6.98%	95.84%/ 7.47%
**Strain**	**BL-S-21**	**BL-S-22**	**BL-S-23**	**BL-S-24**	**BL-S-25**	**BL-S-26**	**BL-S-27**
Genome Size (Mb)	5.89	6.04	6.04	3.96	4.54	4.68	5.18
G + C Content (%)	65.2	64.2	64.2	61.4	66.4	64.3	65.9
Completeness/ Contamination *	97.03%/ 6.48%	97.15%/ 6.92%	97.15%/ 6.73%	93.93%/ 4.19%	99.57%/ 2.30%	99.43%/ 2.26%	99.48%/ 3.72%
**Strain**	**BL-S-29**	**BL-S-30**	**BL-S-31**	**BL-S-32**	**BL-S-33**	**BL-S-34**	
Genome Size (Mb)	5.18	5.07	5.40	5.72	5.77	5.95	
G + C Content (%)	65.9	66.0	65.7	64.0	64.0	65.2	
Completeness/ Contamination *	99.48%/ 3.72%	99.48%/ 3.43%	99.48%/ 3.86%	96.06%/ 6.80%	96.08%/ 6.88%	97.03%/ 7.01%	
**Strain**	**BL-A-28-Hi1-0D**		**BL-A-41-Hi4A-0G**			**BL-A-41-Hi4B-X5A**	
Genome Size (Mb)	4.09		4.50			4.41	
G + C Content (%)	63.8		63.9			63.8	
Completeness/ Contamination *	98.34%/ 1.94%		97.80%/ 2.53%			98.18%/ 2.99%	

* As assessed by CheckM2 (version 1.1.0) [35].

## Data Availability

The stains are stored within the Boudreau Lab culture collection, while the sequence data has been uploaded to GenBank for public archiving. The preliminary 16S rRNA gene sequences were uploaded with the accession numbers: PX452657-PX452709 (see Supplementary Materials Table S3), while the genomes are available under BioProject number PRJNA1357350.

## Supplementary Materials

The following supporting information can be downloaded at: https://www.mdpi.com/article/10.3390/microorganisms14030698/s1, Supplementary File S1: Materials and Methods Supplement: General Experimental Parameters and Materials, Media Preparation, and Methods Supplement on Using the Type (Strain) Genome Server; Table S1: Primers Used to Amplify the Serine Palmitoyltransferase Gene; Table S2: Thermal Cycler PCR Method for the *spt* Gene; Table S3: Summary of 16S rRNA Sequencing Results; Table S4: Initial Flye Genome Assembly Statistics and geNomad Results; Table S5: Summary of the geNomad Plasmid Analysis; Table S6: Summary of geNomad Virus Analysis; Figure S1: Genome phylogeny tree for *Sphingomonas aurantiaca* BL-S-01; Figure S2: Genome phylogeny tree for *Sphingomonas hankookensis* BL-S-02 and *Sphingomonas* sp. BL-S-05. Figure S3: Genome phylogeny tree for *Sphingomonas zeae* BL-S-08 and BL-S-12; Figure S4: Genome phylogeny tree for *Novosphingobium* spp. BL-S-19, BL-S-20, BL-S-21, BL-S-22, BL-S-23, BL-S-32, BL-S-33, and BL-S-34. Figure S5: Genome phylogeny tree for *Novosphingobium* sp. BL-S-24; Figure S6: Genome phylogeny tree for *Sphingomonas* sp. BL-S-25; Figure S7: Genome phylogeny tree for *Sphingomonas bisphenolicum* BL-S-26; Figure S8: Genome phylogeny tree for *Sphingomonas* sp. BL-S-27, BL-S-29, BL-S-30, and BL-S-31; Figure S9: Genome phylogeny tree for *Novosphingobium* spp. BL-A-28-Hi1-0D, BL-A-41-Hi4A-0G, and BL-A-41-Hi4B-X5A; Figure S10: OrthoANI comparison of sequenced genomes assigned as known species against top hits; Figure S11: OrthoANI comparison of plasmids unscored by the geNomad analysis. Supplementary File S2: orthoANI Summary Against the *Sphingomonadaceae*. References [49,50,51,52,53,54,55,56,57,58,59,60,61] are cited in the Supplementary Materials.

## Abbreviations

The following abbreviations are used in this manuscript:

DMS-CA	Defined Medium for Siderophores with Citric Acid
DNA	Deoxyribonucleic Acid
HMW	High Molecular Weight
LB	Luria Broth
OAT	ortho-Average Nucleotide Identity Tool
PBS	Phosphate Buffered Saline
PCR	Polymerase Chain Reaction
SPT	Serine Palmitoyltransferase
STR	Streptomycin
TYGS	Type (Strain) Genome Server
