# Supplementary material for: Novel Genomes of Sphingomonadales Strains Isolated from Diverse Environments
Source: Microorganisms. 2026 Mar 20;14(3):698. doi: 10.3390/microorganisms14030698 (PMC13028669; doi:10.3390/microorganisms14030698)
Supplement: Supplementary file 1 [file microorganisms-14-00698-s001.zip › SI_File S1.pdf]

Article

Serine Palmitoyltransferase Primers Guide Isolation of Sphingomonadales Strains Across Diverse Environments

Nathan W. Williams, Tahir Ali, and Paul D. Boudreau *

Department of BioMolecular Sciences, University of Mississippi School of Pharmacy, University, MS, USA
* Correspondence: boudreau@olemiss.edu

Keywords: Soil Microbiome, Biofilms, Cyanobacterial Microbiome, Whole-Genome Sequencing

Contents

1. Materials and Methods Supplement **2**
1.1. General Experimental Parameters and Materials 2
1.2. Media Preparation 3
1.2.1. Defined Medium for Siderophores with Citric Acid (DMS-CA)[1] . . 3
1.2.2. Luria Broth (LB) 3
1.2.3. Freshwater BG-11 Medium (BG-11) 3
1.3. Methods Supplement on Using the Type (Strain) Genome Server 3
1.3.1. Determination of Closely Related Type Strains 3
1.3.2. Pairwise Comparison of Genome Sequences 4
1.3.3. Phylogenetic Inference 4
1.3.4. Type-based Species and Subspecies Clustering 4

2. 16S Sequencing and Results **4**

3. Genome Assembly Statistics and Annotations **7**

4. Genome and Plasmid Sequence-Based Phylogenies **17**

5. References **28**

check for updates

Received:
Revised:
Accepted:
Published:

Citation: Lastname, F.; Lastname, F.; Lastname, F. Title. *Microorganisms* **2025**, *1*, 0. https://doi.org/

Copyright: © 2025 by the authors. Licensee MDPI, Basel, Switzerland. This article is an open access article distributed under the terms and conditions of the Creative Commons Attribution (CC BY) license (https://creativecommons.org/licenses/by/4.0/).

1. Materials and Methods Supplement

1.1. General Experimental Parameters and Materials

50 mL centrifuge tubes (various suppliers) or Becton Dickinson (Franklin Lakes, NJ, USA) Microbiology Systems culture swabs were used for sample collection. Prepared media and materials were sterilized with an Allen-Bradley autoclave. VWR Life Science (Radnor, PA, USA) bacteriological grade agar was used to solidify media for plates with VWR 100 x 15 mm and Fisher Brand 60 x 15 mm petri dishes. Lab-Line Thermal Rocker (Thermo Scientific; Waltham, MA, USA) was used for gentle mixing and vortexing was accomplished with a Vortex-Genie 2. Tokyo Chemical Industry (Tokyo, Japan) streptomycin sulfate (STR) antibiotic stocks were prepared by passing through Cytiva (Marlborough, MA, USA) Puradisc 13 mm 0.2 µm PES filters using BD 1 mL and 3 mL syringes with Becton Dickinson PrecisionGlide Needle for a 100 mg/mL stock in MilliQ-purified water. 50 mg/L nystatin (Alfa Aesar; Ward Hill, MA, USA) stocks were prepared as a suspension in pure ethanol.

Corning (Tewksbury, MA, USA) LAMBDA Plus Pipettors with Fisherbrand SureOne tips and a Corning STRIPETTOR ULTRA motorized pipet with serological pipets (Vista Labs Technologies; Patterson, NY, USA; or other suppliers) were used for measuring liquid aliquots. Liquid cultures were prepared with Corning 14 mL Polystyrene Round-Bottom Tubes. Incubations were carried out in a VEVOR XHC-25 and a JIUTU JTD-8000 standing incubator, or an Innova 4430 shaking incubator. Samples were handled in a Labconco (Kansas City, MO, USA) Guardian Airflow Monitor biosafety containment system until a preliminary 16S-based identification supported that they were BSL-1 organisms, after which they were handled on the benchtop under flame sterilization. Cryogenic stocks were prepared with Thermo Scientific Nalgene System 100 Cryogenic Tubes, Fisher bioreagents dimethyl sulfoxide (DMSO) ($\geq$97%) or Thermo Scientific glycerol (99+%) as cryopreservants. Storage of samples were in an upright VWR ultralow temperature freezer set for -70 °C.

For PCR, 0.2 mL 8-well PCR Strip tubes (Olympus plastics) were used in combination with an AnaltikJena (Jena, Germany) Biometra TOne, Eppendorf (Hamburg, Germany) Mastercycler nexus GX2e, or Eppendorf Mastercycler nexus gradient thermocycler. For gel electrophoresis, 1x TAE buffer was made from VWR Life Science Tris (Ultra Pure, laboratory use), Fisher Scientific glacial acetic acid (laboratory grade), and EMD Millipore ethylenediaminetetraacetic acid (EDTA) (molecular biology grade) for use with a VWR Mini Electrophoresis System.

A Thermo Scientific Sorvall Legend Micro 21R Centrifuge or Thermo Scientific Sorvall Legend XTR Centrifuge using VWR 1.7 mL microcentrifuge tubes or 50 mL Polypropylene Conical Tubes (Corning or other suppliers), respectively, were used for centrifugation. While a Fisherbrand myspin 6 was used to briefly spin down 1.7 mL tubes or PCR strips.

To isolate genomic DNA, the Omega Bio-Tek E.Z.N.A Bacterial DNA kit and the Macherey-Nagel (Allentown, PA, USA) NucleoBond HMW DNA kit were used. All lysozyme used throughout the experiment was from Omega Bio-Tek (Norcross, GA, USA) E.Z.N.A. Low concentration ($\leq$ 20 ng/µL) low molecular weight DNA samples were reprocessed with the DNA Clean & Concentrator 5 kit (Zymo Research; Irvine, CA, USA) using the vendor's protocol modified to include an extra drying step, a 2 min centrifugation to ensure removal of all alcohol from wash buffers prior to elution. Concentrations were measured with a Qubit 4 Fluorometer (ThermoFisher Scientific) set for 1x dsDNA high sensitivity after samples were prepared with the 1x dsDNA HS Assay Kit (Invitrogen). Samples above the max calibration range were diluted 10x using 180 µL of dsDNA HS Assay Kit buffer and 20 µL of the original sample and remeasured on the Qubit.

1.2. Media Preparation

Media were sterilized by autoclaving using a 30 min liquid cycle. Agar-treated media were poured into sterile plates before solidifying. Use of antimicrobial stocks, nystatin and/or streptomycin sulfate, were added just before pouring. The recipes for the media are detailed below:

1.2.1. Defined Medium for Siderophores with Citric Acid (DMS-CA)[1]

Here citric acid monohydrate was used as the carbon source, as previously explored.[2] Briefly, the medium was prepared with 0.30 g/L KH_2PO_4 (Sigma Aldrich; St. Louis, MO, USA), 0.30 g/L $MgSO_4$ (Sigma-Aldrich), 1.00 g/L citric acid monohydrate (Sigma-Aldrich), 2.00 g/L L-glutamine (Aldrich), 2.00 g/L 3-(*N*-morpholino)propanesulfonic acid (MOPS) (Sigma-Aldrich), and 7 or 15 g/L of agar (VWR Life Science) in ca. 80% total volume of MilliQ water, after being fully dissolved, this mixture was brought to pH 7.2 – 7.5 using 1.0 M aqueous sodium hydroxide, and diluted to the final volume with MilliQ-purified water. Isolates that were isolated on DMS-CA agar plates were grown in a modified liquid Acidovorax Complex Medium (ACM*).[3] This medium differs from DMS-CA by the addition of 0.50 g/L yeast extract (Sigma-Aldrich) and by omitting solidifying agar, but is otherwise prepared in the same fashion.

1.2.2. Luria Broth (LB)

We utilized 20 g/L, of premixed LB Broth Lennox powder (Fisher Bioreagents) in ca. 80% total volume of deionized water with 7 g/L of agar. LB media was then brought to pH of 7.0 using 1.0 M aqueous sodium hydroxide and diluted to the final volume with MilliQ-purified water. A 1/5x dilution was also prepared with 4 g/L instead of 20 g/L and preparation was the same as described for the full-strength LB protocol.

1.2.3. Freshwater BG-11 Medium (BG-11)

This medium was prepared following UTEX Culture Collection of Algae protocol, with the only modification being stocks #5 (citric acid monohydrate) and #6 (ferric ammonium citrate) were prepared as one combined filter-sterilized stock.[4]

1.3. Methods Supplement on Using the Type (Strain) Genome Server

The genome sequence data were uploaded to the Type (Strain) Genome Server (TYGS), a free bioinformatics platform available at https://tygs.dsmz.de, for a whole genome-based taxonomic analysis [5]; with methods details available from the platform provided as follows. The analysis also made use of recently introduced methodological updates and features [6,7]. Information on nomenclature, synonymy and associated taxonomic literature was provided by TYGS's sister database, the List of Prokaryotic names with Standing in Nomenclature (LPSN, available at https://lpsn.dsmz.de) [6,7]. The results were provided by the TYGS on 2025-11-12. Note that initial trees were drawn for each individual strain for preliminary analyses, but the later included figures include trees drawn with closely related strains combined into a single run. The TYGS analysis was subdivided into the following steps:

1.3.1. Determination of Closely Related Type Strains

Determination of closest type strain genomes was done in two complementary ways: First, all user genomes were compared against all type strain genomes available in the TYGS database via the MASH algorithm, a fast approximation of intergenomic relatedness [8], and, the ten type strains with the smallest MASH distances chosen per user genome. Second, an additional set of ten closely related type strains was determined via the 16S rDNA gene sequences. These were extracted from the user genomes using RNAmmer [9]

and each sequence was subsequently BLASTed [10] against the 16S rDNA gene sequence of each of the currently available type strains in the TYGS database. This was used as a proxy to find the best 50 matching type strains (according to the bitscore) for each user genome and to subsequently calculate precise distances using the Genome BLAST Distance Phylogeny approach (GBDP) under the algorithm 'coverage' and distance formula *d5* [11]. These distances were finally used to determine the 10 closest type strain genomes for each of the user genomes.

1.3.2. Pairwise Comparison of Genome Sequences

For the phylogenomic inference, all pairwise comparisons among the set of genomes were conducted using GBDP and accurate intergenomic distances inferred under the algorithm 'trimming' and distance formula *d5* [11]. 100 distance replicates were calculated each. Digital DDH values and confidence intervals were calculated using the recommended settings of the GGDC 4.0 [6,11].

1.3.3. Phylogenetic Inference

The resulting intergenomic distances were used to infer a balanced minimum evolution tree with branch support via FASTME 2.1.6.1 including SPR postprocessing [12]. Branch support was inferred from 100 pseudo-bootstrap replicates each. The trees were rooted at the midpoint [13] and visualized with PhyD3 [14].

1.3.4. Type-based Species and Subspecies Clustering

The type-based species clustering using a 70% dDDH radius around each of the type strains was done as previously described [5]. Subspecies clustering was done using a 79% dDDH threshold as previously introduced [15]. These results are presented in SI Figures S1–S9 with captions detailing the figure generation settings.

2. 16S Sequencing and Results

Table S1. Primers Used to Amplify the *spt* Gene.

Primer Name	Length (bp)	Sequence (5′ to 3′)	Targeted Genus	Reference
PB05	14	ATCCTGCTGGGCAC	*Novosphingobium*	This work
PB07	16	ATCTTGCTTGGCACCT	*Zymomonas*	This work
LT11	14	ATCCTGCTCGGCAC	*Sphingomonas*	[16]
PB06	14	GAGCAGCGCAGCAA	*Novosphingobium*	This work
PB08	16	GAACAACGCAGCAAGA	*Zymomonas*	This work
LT13	14	GAGCAGCGCAGCAG	*Sphingomonas*	[16]

Table S2. Thermal Cycler PCR Method for the *spt* Gene.

Step	Temperature (°C)	Time (s)
1	95	480
2	95	5
3	55	20
4	68	90
Return to Step 2 (x40 cycles)		
5	68	300
6	4	Hold

Table S**3.** Summary of 16S rRNA Sequencing Results, Part 1.

Accession Number	Strain	Length (bp)	No. of Ambiguities	Top BLAST Hit*	WGS?
Files from Automated Trimming (0.5% error probability limit)					
PX452680	**BL-S-05**	1,263	0	*Sphingomonas* sp. HPCWC10 (PP472633)	**Yes**
PX452682	BL-S-07	1,147	0	*S. zeae* 16SBA4 (OR717479)	No
PX452684	BL-S-09	1,149	0	*S. zeae* 16SBA4 (OR717479)	No
PX452687	**BL-S-12**	1,196	0	*S. zeae* 16SBA4 (OR717479)	**Yes**
PX452689	BL-S-14	1,207	0	*S. zeae* 16SBA4 (OR717479)	No
PX452690	BL-S-15	1,205	0	*S. zeae* 16SBA4 (OR717479)	No
PX452691	BL-S-16	1,146	1	*S. zeae* 16SBA4 (OR717479)	No
PX452692	BL-S-17	1,272	0	*S. zeae* 16SBA4 (OR717479)	No
PX452693	BL-S-18	1,308	0	*S. zeae* 16SBA4 (OR717479)	No
PX452694	**BL-S-19**	1,300	1	*N. guangzhouense* HMF7644 (MG383378)	**Yes**
PX452695	**BL-S-20**	1,238	1	*N. lindaniclasticum* Ca-34 (OR781322)	**Yes**
PX452696	**BL-S-21**	1,297	0	*N. lindaniclasticum* YHNG21 (MG516209)	**Yes**
PX452697	**BL-S-22**	1,247	0	*N. guangzhouense* HMF7644 (MG383378)	**Yes**
PX452698	**BL-S-23**	1,300	0	*N. guangzhouense* HMF7644 (MG383378)	**Yes**
PX452699	**BL-S-24**	1,335	0	*N. barchaimii* LD21 (ON738630)	**Yes**
PX452701	**BL-S-26**	1,331	0	*S. bisphenolicum* AO1 (AP018818)	**Yes**
PX452702	**BL-S-27**	1,300	1	*Sphingomonas* sp. GR 6-03 (KM253077)	**Yes**
PX452703	**BL-S-28**	1,283	1	*Sphingomonas* sp. GR 6-03 (KM253077)	**Yes**
PX452704	**BL-S-29**	1,191	1	*Sphingomonas* sp. GR 6-03 (KM253077)	**Yes**
PX452705	**BL-S-30**	1,236	0	*Sphingomonas* sp. GR 6-03 (KM253077)	**Yes**
PX452706	**BL-S-31**	1,238	0	*Sphingomonas* sp. GR 6-03 (KM253077)	**Yes**
PX452707	**BL-S-32**	1,334	0	*N. barchaimii* LD21 (ON738630)	**Yes**
PX452708	**BL-S-33**	1,300	0	*N. barchaimii* LD21 (ON738630)	**Yes**
PX452709	**BL-S-34**	1,323	0	*N. guangzhouense* MB49 (MH675507)	**Yes**
PX452657	BL-A-28-Hi1-0C	1,197	0	*N. taihuense* XDMA_21 (PQ483062)	No
PX452658	**BL-A-28-Hi1-0D**	1,197	0	*N. taihuense* XDMA_21 (PQ483062)	**Yes**
PX452659	BL-A-28-Hi1-0E	1,202	0	*N. taihuense* XDMA_21 (PQ483062)	No
PX452660	BL-A-28-Hi1-X1A	1,385	0	*N. taihuense* XDMA_21 (PQ483062)	No
PX452661	BL-A-28-Hi1-X2A	1,387	0	*N. taihuense* XDMA_21 (PQ483062)	No
PX452662	BL-A-28-Hi1-X3A	1,378	0	*N. taihuense* XDMA_21 (PQ483062)	No
PX452663	BL-A-28-Hi1-X4A	1,350	0	*N. taihuense* XDMA_21 (PQ483062)	No
PX452664	BL-A-28-Hi1-X5A	1,345	0	*N. taihuense* XDMA_21 (PQ483062)	No
PX452665	BL-A-28-Hi1-X6A	1,390	0	*N. taihuense* XDMA_21 (PQ483062)	No
PX452666	BL-A-28-Hi1-X7A	1,359	0	*N. taihuense* XDMA_21 (PQ483062)	No
PX452667	**BL-A-41-Hi4A-0G**	1,160	0	*N. taihuense* XDMA_21 (PQ483062)	**Yes**
PX452668	BL-A-41-Hi4A-0H	1,196	0	*N. taihuense* VNPI57 (OR358898)	No
PX452669	BL-A-41-Hi4A-X1A	1,389	0	*N. taihuense* XDMA_21 (PQ483062)	No
PX452670	BL-A-41-Hi4A-X3A	1,389	0	*N. taihuense* XDMA_21 (PQ483062)	No
PX452671	BL-A-41-Hi4A-X4A	1,373	0	*N. taihuense* XDMA_21 (PQ483062)	No
PX452672	BL-A-41-Hi4A-X6A	1,301	0	*N. taihuense* XDMA_21 (PQ483062)	No
PX452673	BL-A-41-Hi4A-X7A	1,349	0	*N. taihuense* XDMA_21 (PQ483062)	No
PX452674	BL-A-41-Hi4A-X8A	1,334	0	*N. taihuense* XDMA_21 (PQ483062)	No
PX452675	**BL-A-41-Hi4B-X5A**	1,311	0	*N. aromaticivorans* 30-11 (LC875678)	**Yes**

(Continued on the next page.)

*Excludes uncultivated isolates.

Table S3 ***(continued)***. Summary of 16S rRNA Sequencing Results, Part 2.

**Accession Number**	**Strain**	**Length (bp)**	**No. of Ambi-guities**	**Top BLAST Hit***	**WGS?**
Files from Manually Trimmed Alignments					
PX452676	**BL-S-01**	935	2	*Sphingomonas* sp. B2K035a (MN989142)	**Yes**
PX452677	**BL-S-02**	241	2	*S. panni* WSP33-1 (MZ905238)	**Yes**
PX452678	BL-S-03	631	1	*S. hankookensis* M1-1 (KY882114)	No
PX452679	BL-S-04	357	2	*S. hankookensis* B5 (MH605446)	No
PX452681	BL-S-06	719	2	*S. zeae* 16SBA4 (OR717479)	No
PX452683	**BL-S-08**	275	2	*S. aquatilis* LL2H5 (OP080829)	**Yes**
PX452685	BL-S-10	872	2	*S. zeae* 16SBA4 (OR717479)	No
PX452686	BL-S-11	861	2	*S. zeae* 16SBA4 (OR717479)	No
PX452688	BL-S-13	716	2	*S. zeae* 16SBA4 (OR717479)	No
PX452700	**BL-S-25**	891	2	*S. desiccabilis* K13KBY004 (MK106325)	**Yes**

*Excludes uncultivated isolates.

3. Genome Assembly Statistics and Annotations

Table S4. Initial Flye Genome Assembly Statistics and GeNomad Results, Part 1.

Strain	Contig	Contig Length (bp)	Fold Coverage	Circular ? / Repeat? (Y/N)	Multi-plicity	GeNomad Results[a]	
						Plasmid Score	Virus Score
BL-S-01	contig 1	3,884,053	194	Y/N	1	*N/A*	**0.943**[b]
	contig 4	203,518	102	Y/N	1	**0.9867**	*Not Scored*
	contig 5	83,341	173	Y/N	1	**0.9817**	*Not Scored*
	contig 3	16,310	785	Y/Y	4	**0.9936**	*Not Scored*
	contig 2	7,815	2,267	Y/Y	12	**0.9924**	*Not Scored*
BL-S-02	contig 6	3,420,443	362	Y/N	1	*N/A*	**0.9751, 0.9744, 0.9134**[b]
	contig 9	216,849	120	Y/N	1	**0.9859**	*Not Scored*
	contig 4	150,651	190	N/N	1	**0.9896**	*Not Scored*
	contig 5	113,200	149	N/N	1	**0.9926**	*Not Scored*
	contig 12	92,573	177	Y/N	1	*Not Scored*	*Not Scored*
	contig 7	84,774	200	Y/N	1	**0.9843**	*Not Scored*
	contig 1	77,243	348	Y/N	1	**0.9892**	*Not Scored*
	contig 11	49,817	862	Y/Y	2	**0.9929**	*Not Scored*
	contig 8	47,591	591	Y/N	2	**0.9902**	*Not Scored*
	contig 3[c]	11,029	116	N/Y	1	**0.8796**	*Not Scored*
BL-S-05	contig 1	3,780,633	60	Y/N	1	*N/A*	**0.9698, 0.9111, 0.8917**[b]
	contig 4	172,980	107	Y/N	1	**0.9874**	*Not Scored*
	contig 2	134,965	153	Y/N	2	**0.9901**	*Not Scored*
	contig 3	119,826	255	Y/Y	3	*Not Scored*	*Not Scored*
	contig 5	71,704	519	Y/Y	6	**0.9804**	*Not Scored*
	contig 6	63,918	780	Y/Y	10	**0.9907**	*Not Scored*
BL-S-08	contig 3	3,792,792	248	Y/N	1	*N/A*	**0.9972, 0.9529, 0.9466**[b]
	contig 8	206,223	457	Y/N	1	**0.9922**	*Not Scored*
	contig 5	130,903	654	Y/Y	1	*Not Scored*	*Not Scored*
	contig 2	105,049	1,114	Y/Y	2	**0.9891**	*Not Scored*
	contig 7	83,955	879	Y/Y	2	*Not Scored*	*Not Scored*
	contig 4	78,740	2,426	Y/Y	2	**0.9916**	*Not Scored*
	contig 1	57,308	4,319	Y/Y	9	**0.9936**	*Not Scored*
	contig 6	40,068	10,395	Y/Y	22	**0.9932**	*Not Scored*

(Continued on the next page.)

[a]GeNomad analysis performed on the medaka-polished version of the assemblies, not the initial Flye-derived assembly detailed in the other columns of this table.[17,18]

[b]GeNomad virus score(s) for each separate provirus within the chromosome, see Table 6.

[c]This contig excluded from the final assembly.

Table S4 ***(continued)*** Initial Flye Genome Assembly Statistics and GeNomad Results, Part 2.

**Strain**	**Contig**	**Contig Length (bp)**	**Fold Coverage**	**Circular ? / Repeat? (Y/N)**	**Multi-plicity**	**GeNomad Results[a]**	
						**Plasmid Score**	**Virus Score**
BL-S-12	contig 6	3,771,768	208	Y/N	1	*N/A*	**0.9657, 0.9536, 0.9466, 0.9458[b]**
	contig 5	303,359	87	Y/N	1	**0.9894**	*Not Scored*
	contig 13	281,995	74	Y/N	1	**0.9930**	*Not Scored*
	contig 7	275,326	106	Y/N	1	**0.9889**	*Not Scored*
	contig 8	108,380	665	Y/Y	3	**0.9911**	*Not Scored*
	contig 2	104,377	232	Y/N	1	**0.9920**	*Not Scored*
	contig 1[c]	40,100	4,788	N/Y	20	**0.9936**	*Not Scored*
	contig 3[c]	11,672	3,614	N/Y	15	**0.9844**	*Not Scored*
	contig 14	5,657	3,633	Y/Y	15	**0.9947**	*Not Scored*
	contig 12[c]	3,883	70	N/Y	1	*Not Scored*	*Not Scored*
	contig 10[c]	3,070	3,839	N/Y	16	*Not Scored*	*Not Scored*
BL-S-19	contig 2	3,882,746	391	Y/N	1	*N/A*	**0.9654, 0.9544, 0.8506[b]**
	contig 1	2,013,438	360	Y/N	1	**0.9803**	*Not Scored*
	contig 3	52,279	1,673	Y/Y	4	**0.9906**	*Not Scored*
BL-S-20	contig 2	3,285,637	323	Y/N	1	*N/A*	**0.9644, 0.9534, 0.8064[b]**
	contig 1	1,842,723	313	Y/N	1	**0.9802**	*Not Scored*
BL-S-21	contig 3	2,954,539	385	Y/N	1	*N/A*	**0.8646, 0.8335[b]**
	contig 1	2,116,569	392	Y/N	1	**0.9732**	*Not Scored*
	contig 2	819,458	155	Y/N	1	**0.9888**	*Not Scored*
BL-S-22	contig 1	3,957,396	240	Y/N	1	*N/A*	**0.8646, 0.7223[b]**
	contig 2	2,082,338	231	Y/N	1	**0.9828**	*Not Scored*
	contig 4[c]	7,925	116	N/Y	1	*Not Scored*	*Not Scored*
BL-S-23	contig 1	3,957,386	400	Y/N	1	*N/A*	**0.8646, 0.8346[b]**
	contig 2	2,082,336	410	Y/N	1	**0.9827**	*Not Scored*
BL-S-24	contig 2	2,738,720	106	Y/N	1	*N/A*	**0.9698[b]**
	contig 1	1,176,061	103	Y/N	1	**0.9870**	*Not Scored*
	contig 3	47,947	161	Y/N	1	**0.9890**	*Not Scored*
BL-S-25	contig 2	4,537,477	376	Y/N	1	*N/A*	**0.9299[b]**

(Continued on the next page.)

[a]GeNomad analysis performed on the medaka-polished version of the assemblies, not the initial Flye-derived assembly detailed in the other columns of this table.[17,18]

[b]GeNomad virus score(s) for each separate provirus within the chromosome, see Table 6.

[c]This contig excluded from the final assembly.

Table S4 ***(continued)*** Initial Flye Genome Assembly Statistics and GeNomad Results, Part 3.

Strain	Contig	Contig Length (bp)	Fold Coverage	Circular ? / Repeat? (Y/N)	Multi-plicity	GeNomad Results[a]	
						Plasmid Score	Virus Score
BL-S-26	contig 2	3,731,618	728	Y/N	1	*N/A*	**0.9551, 0.9546, 0.9523, 0.9185, 0.8541**[b]
	contig 1	851,238	758	Y/N	1	**0.9881**	*Not Scored*
	contig 3	102,328	1,268	Y/N	1	**0.9924**	*Not Scored*
BL-S-27	contig 1	5,179,297	184	Y/N	1	*N/A*	*Not Scored*
BL-S-28[c]	contig 2	3,957,384	482	Y/N	1	*N/A*	**0.9225, 0.8535**[b]
	contig 1	2,082,338	478	Y/N	1	**0.9831**	*Not Scored*
BL-S-29	contig 1	5,179,301	430	Y/Y	1	*N/A*	*Not Scored*
	contig 2[d]	5,766	142	N/Y	142	*Not Scored*	*Not Scored*
	contig 3[d]	5,551	94	N/Y	1	*Not Scored*	*Not Scored*
BL-S-30	contig 1	5,070,176	443	Y/N	1	*N/A*	*Not Scored*
BL-S-31	contig 2	5,249,461	333	N/N	1	*N/A*	**0.9735**[b]
	contig 3	155,136	284	N/N	1	**0.9879**	*Not Scored*
	contig 1[d]	24,840	99	N/N	1	**0.9592**	*Not Scored*
BL-S-32	contig 1	3,606,184	34	Y/N	1	*N/A*	**0.9287**[b]
	contig 2	2,110,857	30	Y/N	1	**0.9849**	*Not Scored*
BL-S-33	contig 2	3,678,722	284	Y/N	1	*N/A*	*Not Scored*
	contig 1	2,088,711	214	Y/N	1	**0.9859**	*Not Scored*
BL-S-34	contig 3	3,019,779	37	Y/N	1	*N/A*	**0.8447, 0.8299**[b]
	contig 1	2,097,549	40	Y/N	1	**0.9738**	*Not Scored*
	contig 2	838,476	18	Y/N	1	**0.9893**	*Not Scored*
BL-A-28-Hi1-0D	contig 2	3,679,240	53	Y/N	1	*N/A*	**0.9409**[b]
	contig 1	407,646	62	Y/N	1	**0.9877**	*Not Scored*
BL-A-41-Hi4A-0G	contig 4	3,638,869	205	Y/N	1	*N/A*	**0.9476**[b]
	contig 1	469,182	214	Y/N	1	**0.9889**	*Not Scored*
	contig 2	206,367	278	Y/N	1	**0.9907**	*Not Scored*
	contig 3	185,983	293	Y/N	1	**0.9872**	*Not Scored*
BL-A-41-Hi4B-X5A	contig 4	3,233,879	209	Y/N	1	*N/A*	**0.9705, 0.954, 0.9415**[b]
	contig 1	878,784	237	Y/N	1	**0.9892**	*Not Scored*
	contig 2	184,700	320	Y/N	1	**0.9903**	*Not Scored*
	contig 3	117,026	284	Y/N	1	**0.9893**	*Not Scored*

[a]GeNomad analysis performed on the medaka-polished version of the assemblies, not the initial Flye-derived assembly detailed in the other columns of this table.[17,18]

[b]GeNomad virus score(s) for each separate provirus within the chromosome, see Table 6.

[c]This genome's 16S gene sequence did not match to the original 16S gene sequence (PX452703), suggesting contamination of the culture used to prepare HMW DNA.

[d]This contig excluded from the final assembly.

Table S5. Summary of GeNomad Plasmid Analysis, Part 1.

Strain	Contig	Plasmid Length (bp)	No. of Genes	Plasmid Score	No. of Hall-marks	Marker Enrich-ment	Conjugation Genes	AMR Genes
BL-S-01	contig 4	203,518	187	**0.9867**	11	48.1825	F_traG, F_traH, F_traF, F_trbC, F_trbC, F_traU, F_traW, F_traV	*Not found*
	contig 5	83,341	78	**0.9817**	3	1.8653	*Not found*	*Not found*
	contig 3[a]	16,310	21	**0.9936**	2	4.6198	*Not found*	*Not found*
	contig 2	7,815	13	**0.9924**	1	5.7018	*Not found*	*Not found*
BL-S-02	contig 9	216,845	213	**0.9859**	1	30.9004	*Not found*	*Not found*
	contig 4	150,651	157	**0.9896**	1	61.6093	*Not found*	*Not found*
	contig 5	113,200	126	**0.9926**	14	59.8267	MOBP1,T_virB9, T_virB8, T_virb6, T_virB5, virB4, T_virB3, T_virB2	*Not found*
	contig 7	84,773	84	**0.9843**	1	10.8774	*Not found*	NF033075
	contig 1	77,243	71	**0.9892**	0	10.6432	*Not found*	*Not found*
	contig 11	49,823	57	**0.9929**	6	13.0369	*Not found*	*Not found*
	contig 8	47,591	50	**0.9902**	0	5.3428	*Not found*	*Not found*
	contig 3[b]	11,029	13	**0.8796**	0	2.0987	*Not found*	*Not found*
BL-S-05	contig 2	134,963	142	**0.9901**	5	43.6392	t4cp2, F_traG, F_traH, F_traF	*Not found*
	contig 3	119,825	101	**0.9874**	1	7.9064	*Not found*	NF033105, NF033105
	contig 5	71,703	58	**0.9804**	1	7.11	*Not found*	*Not found*
	contig 6	63,918	73	**0.9907**	1	22.4632	*Not found*	NF000272
BL-S-08	contig 8	206,223	196	**0.9922**	3	46.7132	t4cp2	*Not found*
	contig 2	105,049	110	**0.9891**	0	31.4913	*Not found*	*Not found*
	contig 4	78,740	80	**0.9916**	2	23.1686	*Not found*	*Not found*
	contig 1	57,308	59	**0.9936**	16	35.6924	F_traG, F_traH, F_traF, F_trbC, F_trbC, F_traU, F_traW, F_traV, F_traK, F_traE, F_traL	*Not found*
	contig 6	40,068	46	**0.9932**	3	21.827	*Not found*	NF000272

(Continued on the next page.)

Abbreviation used: Anti-Microbial Resistance (AMR)

GeNomad scored all plasmids sequences here as Genetic Code = 11 (standard code for Bacteria and Archaea). The false discovery rate was not calculated.

[a] The topology of all plasmids was found to be no terminal repeats, except this contig which was annotated as DTR (direct terminal repeats).

[b] This contig was excluded from the final assembly.

Table S5 ***(continued)*** Summary of GeNomad Plasmid Analysis, Part 2.

Strain	Contig	Plasmid Length (bp)	No. of Genes	Plasmid Score	No. of Hall-marks	Marker Enrich-ment	Conjugation Genes	AMR Genes
BL-S-12	contig 5	303,361	317	**0.9894**	19	111.1429	t4cp2, F_traG, F_traH, F_traF, F_trbC, F_trbC, F_traU, F_traW, F_traV, F_traK, F_traE, F_traL, F_traE	*Not found*
	contig 13	281,995	295	**0.9930**	19	98.4520	F_traL, F_traE, F_traK, F_traV, F_traW, F_traU, F_trbC, F_trbC, F_traF, F_traH, F_traG, F_traE, F_traE, t4cp2	NF000272, NF033105
	contig 7	275,268	294	**0.9889**	20	97.9936	F_traE, F_traG, F_traH, F_traF, F_trbC, F_trbC, F_traU, F_traW, F_traV, F_traK, F_traE, F_traL	NF000272, NF033105
	contig 8	108,380	125	**0.9911**	16	66.2253	F_traL, F_traE, F_traK, F_traV, F_traW, F_traU, F_trbC, F_trbC, F_traF, F_traH, F_traG	*Not found*
	contig 2	104,376	93	**0.9920**	2	16.3445	*Not found*	*Not found*
	contig 1[a]	40,085	46	**0.9936**	3	21.8270	*Not found*	NF000272
	contig 3[a]	11,672	12	**0.9844**	1	4.8613	*Not found*	NF000272
	contig 14	5,657	10	**0.9947**	1	6.6446	*Not found*	*Not found*
BL-S-19	contig 1	2,013,439	1,644	**0.9803**	20	83.305	F_traE, T_virB11, T_virB2, T_virB3, virb4, T_virB5, T_virB6, T_virB8, T_virB9, T_virB10, T_virB10, T_virB9, T_virB8, T_virB6, T_virB5, virb4, T_virB3, T_virB2, T_virB11	NF000272, NF000496, NF033105, NF033068, NF033105, NF000493, NF000088, NF012178, NF000025, NF033135
	contig 3	52,278	50	**0.9906**	2	2.8857	F_traE	*Not found*
BL-S-20	contig 1	1,842,724	1,493	**0.9802**	7	69.4876	T_virB11, F_traE, t4cp2, T_virB11, T_virB8	NF000496, NF000402, NF033135, NF000496, NF000272, NF000237, NF000402, NF033145

(Continued on the next page.)

Abbreviation used: Anti-Microbial Resistance (AMR).

GeNomad scored all plasmids sequences here as Genetic Code = 11 (standard code for Bacteria and Archaea). The false discovery rate was not calculated. The topology of all plasmids was found to be no terminal repeats.

[a]This contig was excluded from the final assembly.

Table S5 ***(continued)*** Summary of GeNomad Plasmid Analysis, Part 3.

Strain	Contig	Plasmid Length (bp)	No. of Genes	Plasmid Score	No. of Hall-marks	Marker Enrich-ment	Conjugation Genes	AMR Genes
BL-S-21	contig 1	2,116,569	1,659	**0.9732**	11	43.431	T_virB11, T_virB10, T_virB9, T_virB8, T_virB6, T_virB5, virb4, T_virB3, T_virB2, T_virB11, F_traE	NF000005, NF000272, NF000496, NF033117, NF000496, NF012174, NF000496, NF000095, NF033145, NF033135, NF000237, NF000402, NF033135, NF000402, NF000496
	contig 2	819,459	704	**0.9888**	11	83.1272	T_virB11, T_virB2, T_virB3, virb4, T_virB5, T_virB6, T_virB8, T_virB9, T_virB10, F_traE	NF000088
BL-S-22	contig 2	2,082,338	1,709	**0.9828**	19	79.024	T_virB10, T_virB9, T_virB8, T_virB6, T_virB5, virb4, T_virB3, T_virB2, T_virB11, T_virB11, T_virB2, T_virB3, virb4, T_virB5, T_virB6, T_virB8, T_virB9, T_virB10	NF000496, NF000272, NF033088, NF033135, NF000025, NF012178, NF000088, NF033105
BL-S-23	contig 2	2,082,337	1,710	**0.9827**	19	78.0995	T_virB10, T_virB9, T_virB8, T_virB6, T_virB5, virb4, T_virB3, T_virB2, T_virB11, T_virB11, T_virB2, T_virB3, virb4, T_virB5, T_virB6, T_virB8, T_virB9, T_virB10	NF000025, NF012178, NF000088, NF033105, NF000496, NF000272, NF033088, NF033135
BL-S-24	contig 1	1,176,060	998	**0.9870**	1	52.7146	MOBQ	NF012174, NF033145, NF000496, NF000496, NF033135, NF012171, NF012171, NF000406, NF0004025
	contig 3	47,947	46	**0.9890**	3	6.9603	*Not found*	*Not found*

(Continued on the next page.)

Abbreviation used: Anti-Microbial Resistance (AMR).

GeNomad scored all plasmids sequences here as Genetic Code = 11 (standard code for Bacteria and Archaea). The false discovery rate was not calculated. The topology of all plasmids was found to be no terminal repeats.

Table S5 ***(continued)*** Summary of GeNomad Plasmid Analysis, Part 4.

Strain	Contig	Plasmid Length (bp)	No. of Genes	Plasmid Score	No. of Hall-marks	Marker Enrich-ment	Conjugation Genes	AMR Genes
BL-S-26	contig 1	851,240	719	**0.9881**	0	72.2576	*Not found*	NF000272, NF033135, NF012174, NF033135
	contig 3	102,328	106	**0.9924**	12	51.7401	MOBP1, T_virB9, T_virB8 , T_virB6, T_virB5, virb4, T_virB3, T_virB2, T_virB1	NF000272
BL-S-28[a]	contig 1	2,082,338	1,709	**0.9831**	19	79.0240	T_virB10, T_virB9, T_virB8, T_virB6, T_virB5, virb4; T_virB3, T_virB2, T_virB11, T_virB11, T_virB2, T_virB3, virb4, T_virB5, T_virB6, T_virB8, T_virB9, T_virB10	NF033105, NF000088, NF012178, NF000025, NF033135, NF033088, NF000272, NF000496
BL-S-31	contig 3	155,129	138	**0.9879**	13	38.3803	F_traL, F_traE, F_traK, F_traV, virb4, F_traW, F_traU, F_trbC, F_trbC, F_traH	NF012174
	contig 1[b]	24,840	25	**0.9592**	0	3.4491	*Not found*	*Not found*
BL-S-32	contig 2	2,110,855	1,743	**0.9849**	29	121.6818	F_traE, F_traE, T_virB10, T_virB9, T_virB8, T_virB6, T_virB5, virb4, T_virB3, T_virB2, T_virB11, B_traH, T_virB11, T_virB3, virb4, T_virB6, T_virB8, T_virB10, F_traE, F_traE, T_virB11, T_virB2, T_virB3, virb4, T_virB5, T_virB6, T_virB8, T_virB9, T_virB10, F_traE	NF000088, NF012178, NF033105, NF000496, NF033134

(Continued on the next page.)

Abbreviation used: Anti-Microbial Resistance (AMR).

GeNomad scored all plasmids sequences here as Genetic Code = 11 (standard code for Bacteria and Archaea). The false discovery rate was not calculated. The topology of all plasmids was found to be no terminal repeats.

[a]This genome's 16S gene sequence did not match to the original 16S gene sequence (PX452703), suggesting contamination of the culture used to prepare HMW DNA.

[b]This contig was excluded from the final assembly.

Table S**5** ***(continued)*** Summary of GeNomad Plasmid Analysis, Part 5.

Strain	Contig	Plasmid Length (bp)	No. of Genes	Plasmid Score	No. of Hall-marks	Marker Enrich-ment	Conjugation Genes	AMR Genes
BL-S-33	contig 1	2,088,715	1,690	**0.9859**	14	112.5001	T_virB10, T_virB9, T_virB8, T_virB6, T_virB5, virb4, T_virB3, T_virB2, T_virB11, F_traE, T_virB11, T_virB8, T_virB9, T_virB10	NF000088, NF012178, NF000493
BL-A-28-Hi1-0D	contig 1	407,646	374	**0.9877**	0	36.7639	*Not found*	NF033088, NF000496
BL-A-41-Hi4A-0G	contig 1	469,182	422	**0.9889**	1	41.4493	T_virB9	NF033088, NF000496, NF033135
	contig 2	206,367	202	**0.9907**	4	55.9507	F_traE, t4cp2	NF012178, NF000272
	contig 3	185,983	186	**0.9872**	16	65.5894	F_traL, F_traE, F_traK, F_traW, F_traU, F_trbC, F_trbC, F_traF, F_traH, F_traG, t4cp2	*Not found*
BL-A-41-Hi4B-X5A	contig 1	878,784	760	**0.9892**	2	78.7049	T_virB9	NF000496, NF000496, NF033135, NF033105, NF033145, NF033088, NF000272, NF033105
	contig 2	184,700	187	**0.9903**	13	68.2648	T_virB9, T_virB8, T_virB6, T_virB5, virb4, T_virB3, T_virB2, T_virB1, MOBP1	NF000272
	contig 3	117,026	115	**0.9893**	4	47.443	F_traF, F_traH, F_traG; t4cp2	*Not found*

Abbreviation used: Anti-Microbial Resistance (AMR).

GeNomad scored all plasmids sequences here as Genetic Code = 11 (standard code for Bacteria and Archaea). The false discovery rate was not calculated. The topology of all plasmids was found to be no terminal repeats.

Table S**6.** Summary of GeNomad Virus Analysis, Part 1.

Strain	Contig	Virus Length (bp)	Topology	Coordinates	No. of Genes	Virus Score	No. of Hall-marks	Marker Enrich-ment
BL-S-01	contig 1	39,292	Provirus	716,642 - 755,933	42	**0.943**	4	24.5359
BL-S-02	contig 6	10,466	Provirus	255,152 - 265,617	13	**0.9134**	8	14.1319
	contig 6	42,473	Provirus	890,903 - 933,375	50	**0.9744**	10	42.2798
	contig 6	41,513	Provirus	1,574,306 - 1,615,818	46	**0.9751**	7	39.0359
BL-S-05	contig 1	43,215	Provirus	1,195,608 - 1,238,822	62	**0.9111**	3	9.0222
	contig 1	31,984	Provirus	1,467,686 - 1,499,669	38	**0.9698**	4	15.6827
	contig 1	15,937	Provirus	2,897,303 - 2,913,239	22	**0.8917**	9	18.8595
BL-S-08	contig 3	38,234	Provirus	419,111 - 457,344	49	**0.9529**	8	34.1097
	contig 3	13,432	Provirus	1,646,884 - 1,660,315	17	**0.9466**	11	21.7694
	contig 3	42,700	Provirus	3,582,451 - 3,625,150	63	**0.9772**	10	47.4908
BL-S-12	contig 6	68,324	Provirus	130,605 - 198,928	104	**0.9458**	17	42.7056
	contig 6	41,691	Provirus	1,420,180 - 1,461,870	51	**0.9657**	3	28.7666
	contig 6	36,412	Provirus	3,060,596 - 3,097,007	57	**0.9536**	16	46.1484
	contig 6	13,432	Provirus	3,719,392 - 3,732,823	17	**0.9466**	11	21.7694
BL-S-19	contig 2	33,762	Provirus	2,276,627 - 2,310,388	40	**0.9544**	8	26.4565
	contig 2	38,602	Provirus	2,861,222 - 2,899,823	43	**0.9654**	11	31.2681
	contig 2	16,357	Provirus	3,806,412-3,822,768	20	**0.8506**	10	22.2843
BL-S-20	contig 2	16,015	Provirus	335,035-351,049	20	**0.8064**	10	22.2843
	contig 2	95,687	Provirus	857,239-952,925	136	**0.9644**	16	62.5283
	contig 2	43,868	Provirus	2,267,140-2,311,007	55	**0.9534**	12	35.8575
BL-S-21	contig 3	16,302	Provirus	1,781,945 - 1,798,246	20	**0.8335**	10	21.0022
BL-S-22	contig 1	16,346	Provirus	65,769-82,114	20	**0.8646**	10	22.2843
	contig 1	24,659	Provirus	1,599,501-1,624,159	31	**0.7223**	0	5.8523
BL-S-23	contig 1	16,346	Provirus	51,553-67,898	20	**0.8646**	10	22.2843
	contig 1	24,676	Provirus	1,585,271-1,609,946	29	0.8346	0	5.8523
BL-S-24	contig 2	16,134	Provirus	1,465,549-1,481,682	20	**0.9359**	12	23.7203
BL-S-25	contig 1	22,475	Provirus	3,056,996-3,079,470	24	**0.9299**	4	19.3097
BL-S-26	contig 2	41,408	Provirus	3,614,397 - 3,655,804	52	**0.9551**	11	38.5269
	contig 2	48,938	Provirus	2,132,949 - 2,181,886	70	**0.9546**	10	47.0913
	contig 2	34,934	Provirus	1,011,759 - 1,046,692	46	**0.9523**	17	47.3948
	contig 2	44,126	Provirus	1,768,933 - 1,813,058	54	**0.9185**	15	42.7910
	contig 2	31,945	Provirus	2,859,602 - 2,891,546	40	**0.8541**	11	25.7547
BL-S-28[a]	contig 2	16,346	Provirus	365,520-381,865	20	**0.8535**	10	22.2843
	contig 2	20,874	Provirus	2,784,645-2,805,518	28	**0.9225**	0	7.5894
BL-S-31	contig 2	26,570	Provirus	3,973,264 - 3,999,833	37	**0.9735**	5	30.6912
BL-S-32	contig 1	43,795	Provirus	2,571,974-2,615,768	60	**0.9287**	16	43.6486
BL-S-34	contig 3	16,178	Provirus	1,350,106 - 1,366,283	23	**0.8447**	1	9.3000
	contig 3	16,302	Provirus	1,774,403 - 1,790,704	19	**0.8299**	10	21.0022

GeNomad scored all provirus sequences as Genetic Code = 11 (standard code for Bacteria and Archaea). The false discovery rate was not calculated. The assessed viral taxonomy for all of the provirus sequences was: Duplodnaviria, Heunggongvirae, Uroviricota, Caudoviricetes. Note that strains BL-S-27, BL-S-29, BL-S-30, and BL-S-33 had no viral hits.

[a] This genome's 16S gene sequence did not match to the original 16S gene sequence (PX452703), suggesting contamination of the culture used to prepare HMW DNA.

(Continued on the next page.)

Table S6 ***(continued)*** Summary of GeNomad[18] Virus Analysis, Part 2.

Strain	Contig	Virus Length (bp)	Topology	Coordinates	No. of Genes	Virus Score	No. of Hall-marks	Marker Enrich-ment
BL-A-28-Hi1-0D	contig 2	15,381	Provirus	747,080 - 762,460	19	**0.9409**	10	20.5752
BL-A-41-Hi4A-0G	contig 4	41,542	Provirus	1,223,333 - 1,264,874	61	**0.9476**	12	35.0337
BL-A-41-Hi4B-X5A	contig 4	42,746	Provirus	1,618,116 - 1,660,861	59	**0.9705**	15	43.5349
	contig 4	15,355	Provirus	2,939,692 - 2,955,046	19	**0.9415**	10	20.5752
	contig 4	34,358	Provirus	2,984,753 - 3,019,110	48	**0.954**	19	39.071

GeNomad scored all provirus sequences as Genetic Code = 11 (standard code for Bacteria and Archaea). The false discovery rate was not calculated. The assessed viral taxonomy for all of the provirus sequences was: Duplodnaviria, Heunggongvirae, Uroviricota, Caudoviricetes. Note that strains BL-S-27, BL-S-29, BL-S-30, and BL-S-33 had no viral hits.

4. Genome and Plasmid Sequence-Based Phylogenies

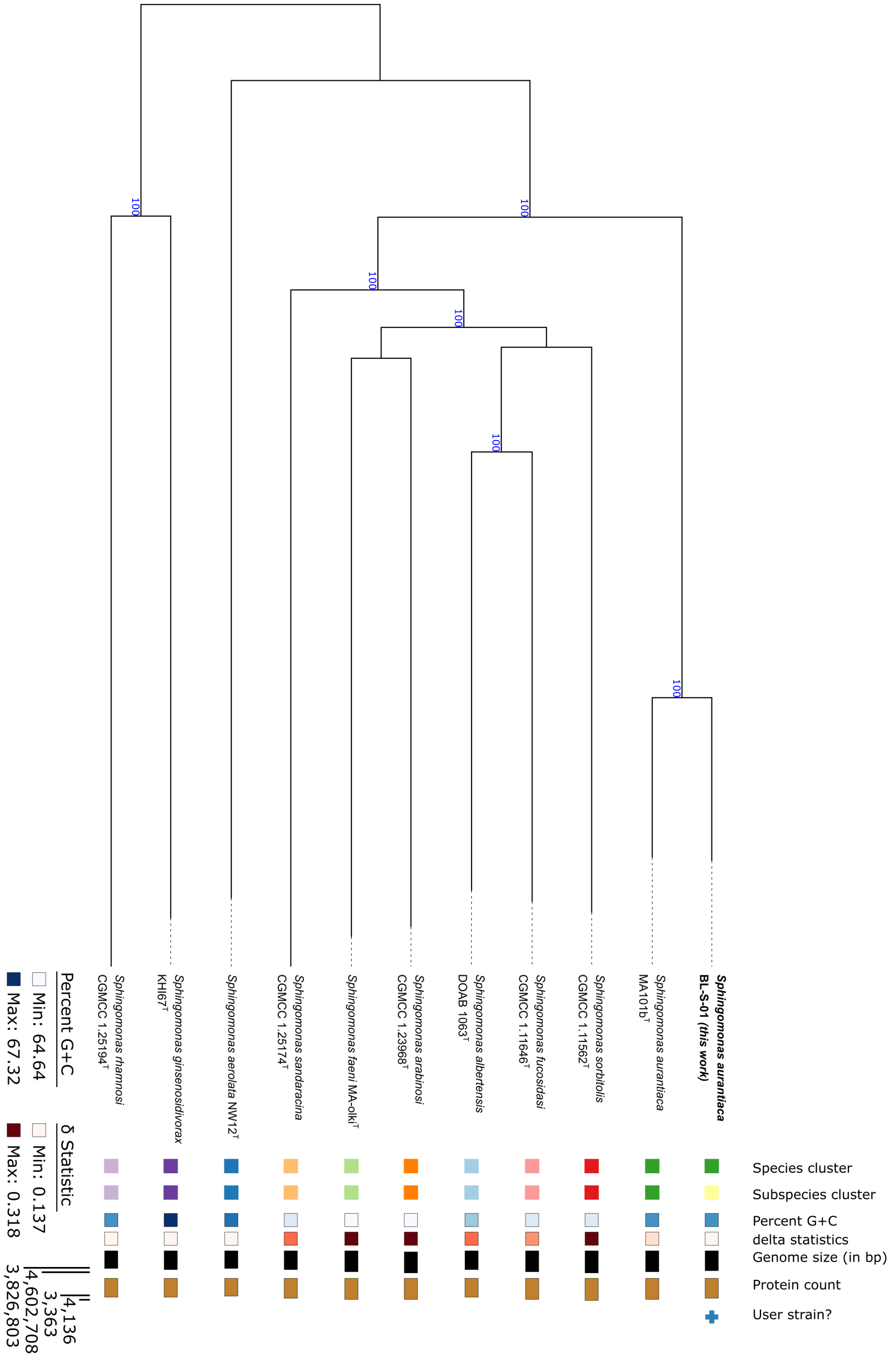

Figure S1. Genome phylogeny tree for *Sphingomonas aurantiaca* BL-S-01. Genome-based tree was made using the TYGS platform, with additional labels appended for clarity. The distance formula was D5 and the distance algorithm was GreedyWithTrimming. Tree inferred with FastME 2.1.6.1 [12] from GBDP (Genome BLAST Distance Phylogeny) distances calculated from genome sequences. The numbers above branches are GBDP pseudo-bootstrap support values >60% from 100 replications (represented in blue numbers), with an average branch support of 89.0%. The tree was rooted at the midpoint [13].

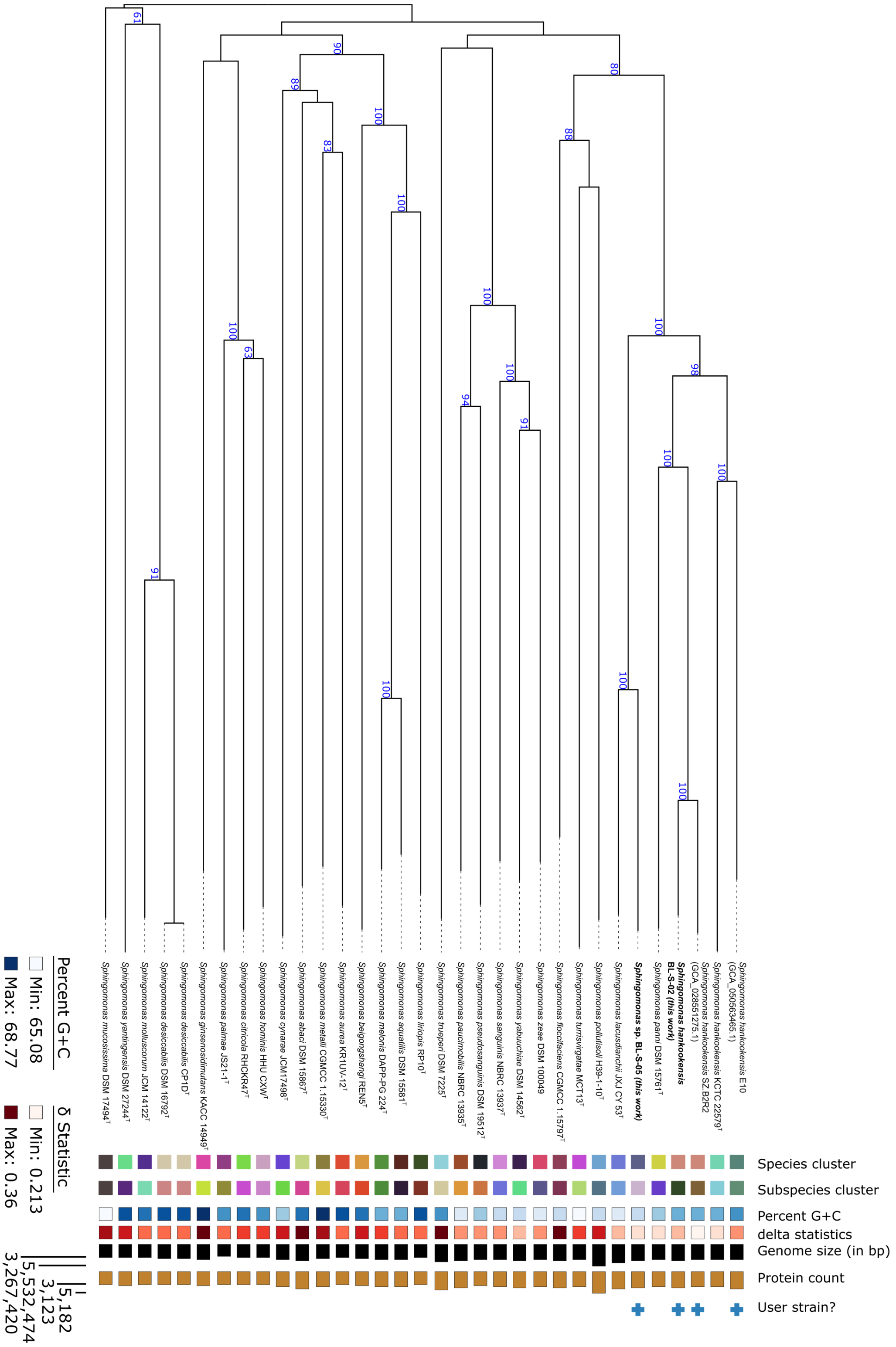

Figure S2. Genome phylogeny tree for *Sphingomonas hankookensis* BL-S-02 and *Sphingomonas* sp. BL-S-05. Two strains of *S. hankookensis* (SZ.B2R2 and E10) were added to the analysis manually. Genome-based tree was made using the TYGS platform, with additional labels appended for clarity. The distance formula was D5 and the distance algorithm was GreedyWithTrimming. Tree inferred with FastME 2.1.6.1 [12] from GBDP (Genome BLAST Distance Phylogeny) distances calculated from genome sequences. The numbers above branches are GBDP pseudo-bootstrap support values >60% from 100 replications (represented in blue numbers), with an average branch support of 79.7%. The tree was rooted at the midpoint [13].

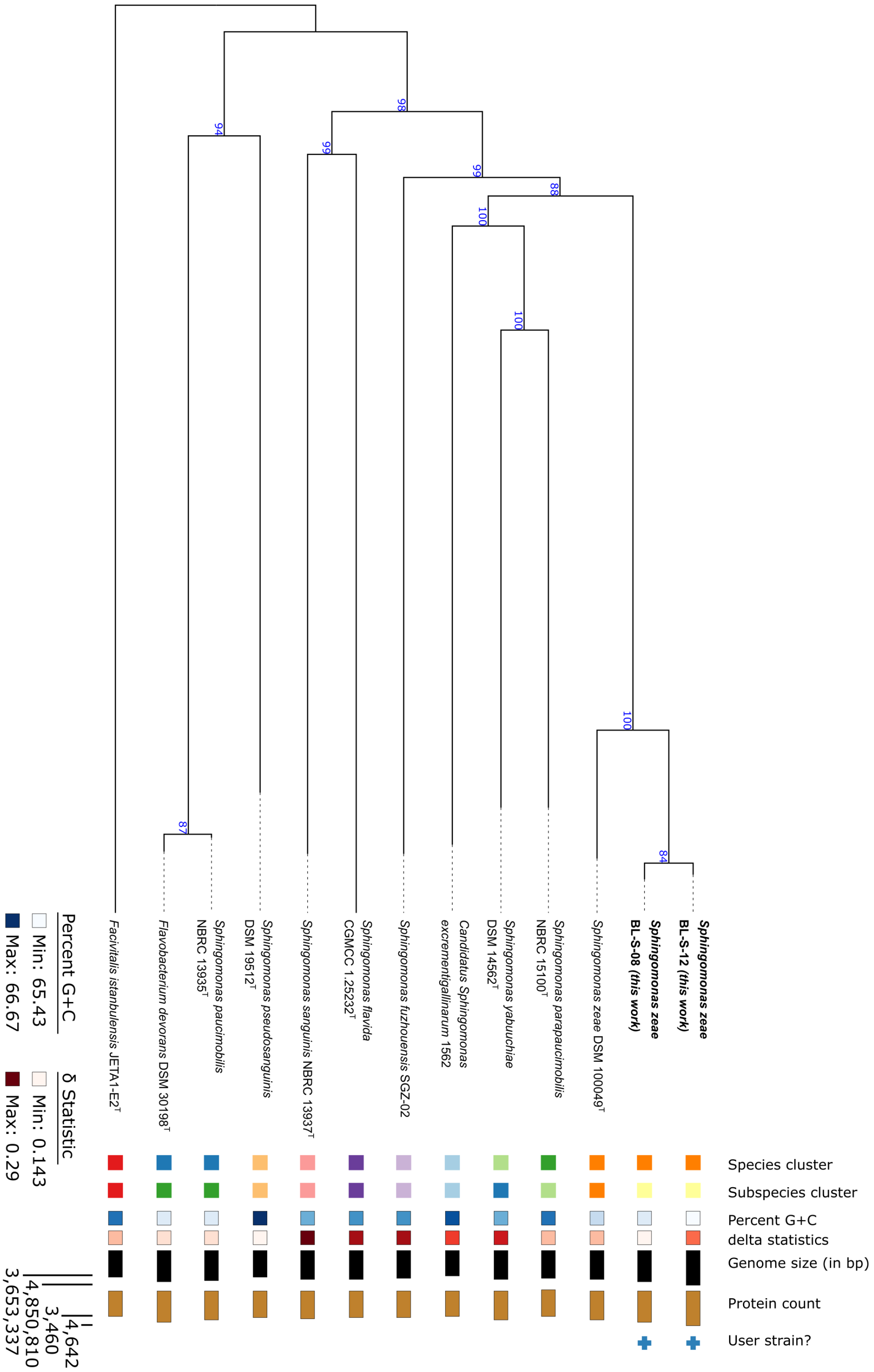

Figure S**3.** Genome phylogeny tree for *Sphingomonas zeae* BL-S-08 and BL-S-12. Genome-based tree was made using the TYGS platform, with additional labels appended for clarity. The distance formula was D5 and the distance algorithm was GreedyWithTrimming. Tree inferred with FastME 2.1.6.1 [12] from GBDP (Genome BLAST Distance Phylogeny) distances calculated from genome sequences. The numbers above branches are GBDP pseudo-bootstrap support values >60% from 100 replications (represented in blue numbers), with an average branch support of 94.9%. The tree was rooted at the midpoint [13].

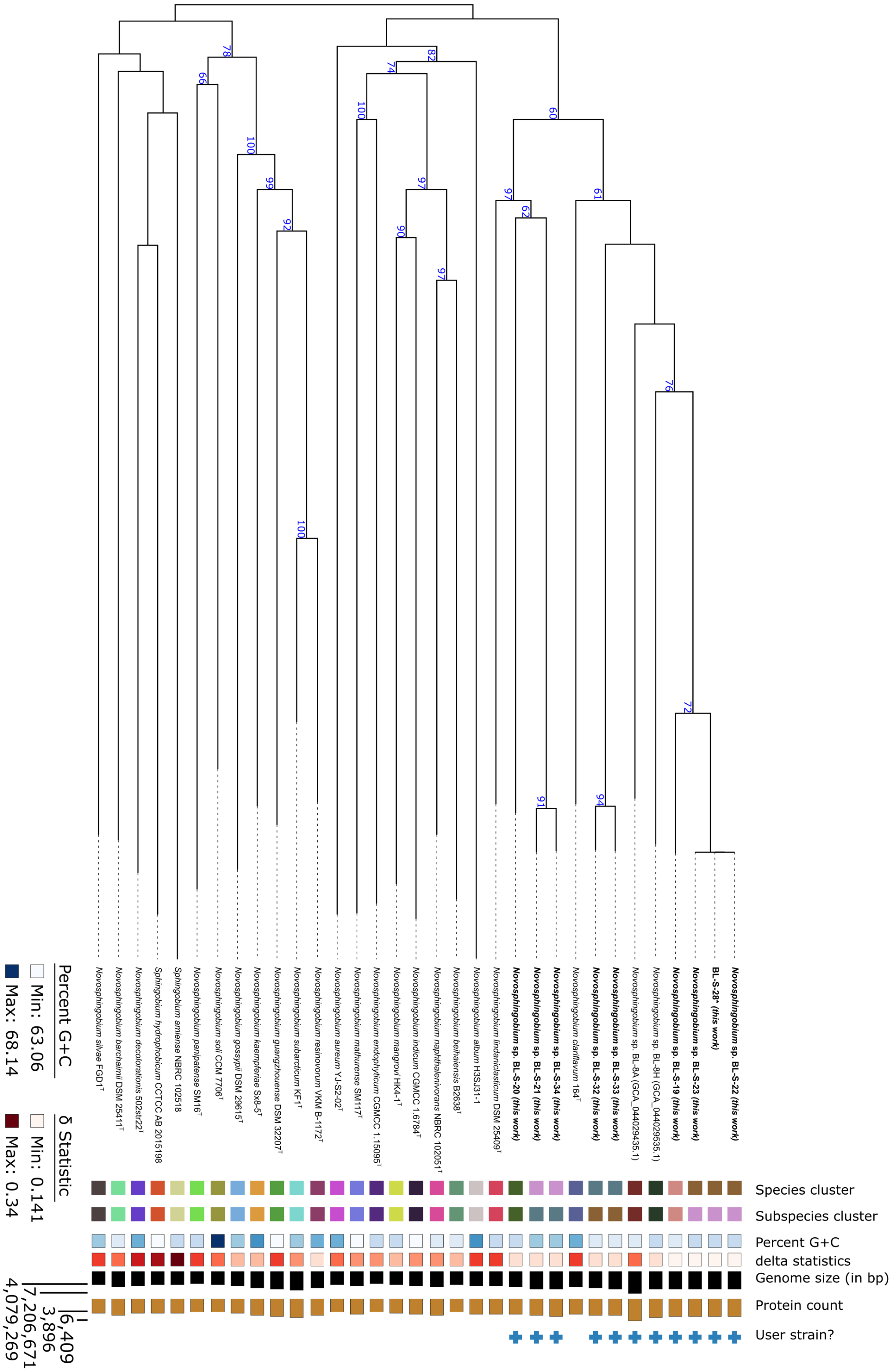

Figure S4. Genome phylogeny tree for *Novosphingobium* spp. BL-S-19, BL-S-20, BL-S-21, BL-S-22, BL-S-23, BL-S-32, BL-S-33, and BL-S-34. Two strains of *Novosphingobium* (BL-8A and BL-8H) were added to the analysis manually. Genome-based tree was made using the TYGS platform, with additional labels appended for clarity. The distance formula was D5 and the distance algorithm was GreedyWithTrimming. Tree inferred with FastME 2.1.6.1 [12] from GBDP (Genome BLAST Distance Phylogeny) distances calculated from genome sequences. The numbers above branches are GBDP pseudo-bootstrap support values >60% from 100 replications (represented in blue numbers), with an average branch support of 70.0%. *This genome does not clade as predicted by the original *Sphingomonas*-like 16S gene sequence (PX452703), suggesting contamination of the culture used to prepare HMW DNA. The tree was rooted at the midpoint [13].

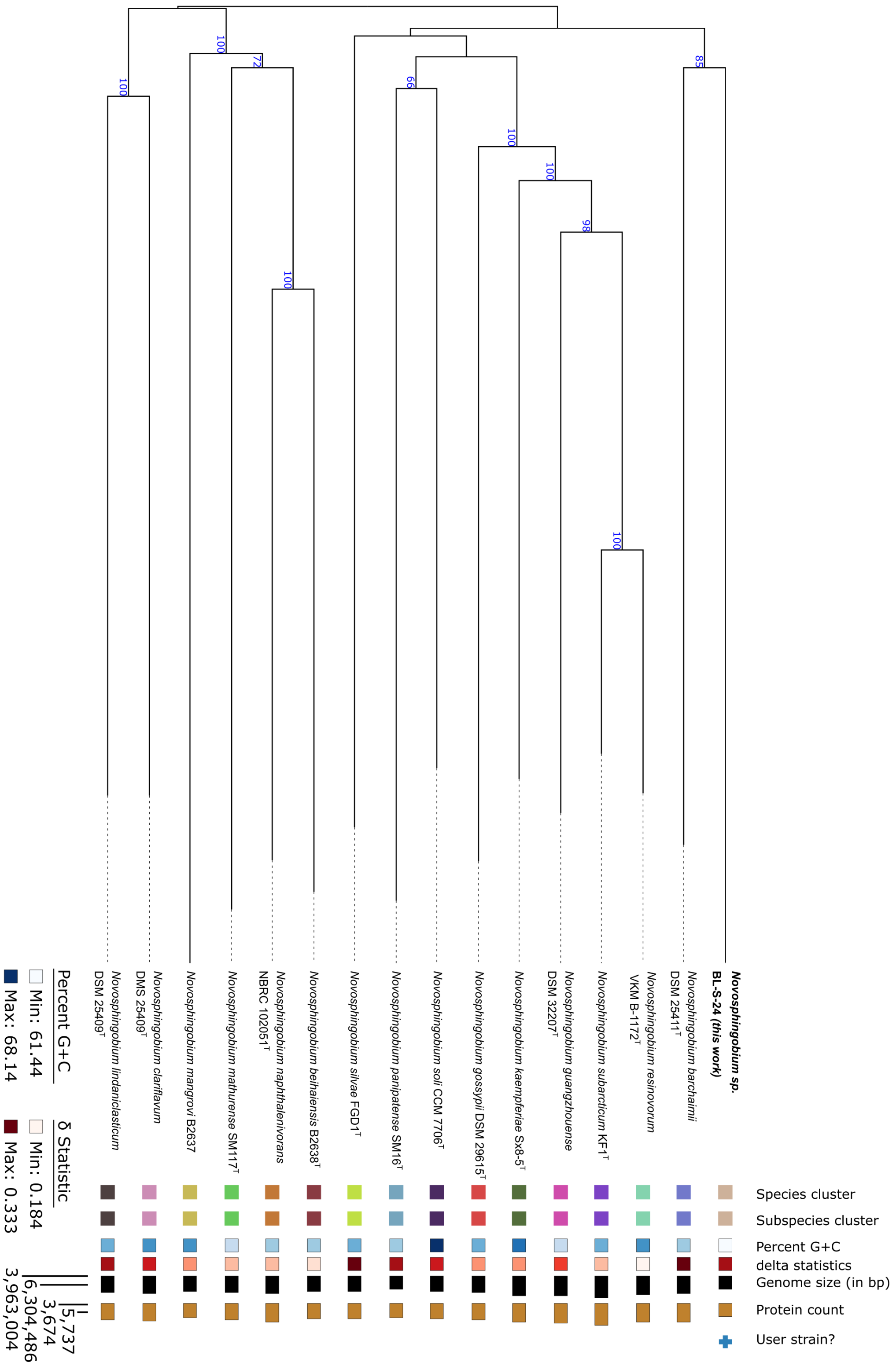

Figure S5. Genome phylogeny tree for *Novosphingobium* sp. BL-S-24. Genome-based tree was made using the TYGS platform, with additional labels appended for clarity. The distance formula was D5 and the distance algorithm was GreedyWithTrimming. Tree inferred with FastME 2.1.6.1 [12] from GBDP (Genome BLAST Distance Phylogeny) distances calculated from genome sequences. The numbers above branches are GBDP pseudo-bootstrap support values >60% from 100 replications (represented in blue numbers), with an average branch support of 83.8%. The tree was rooted at the midpoint [13].

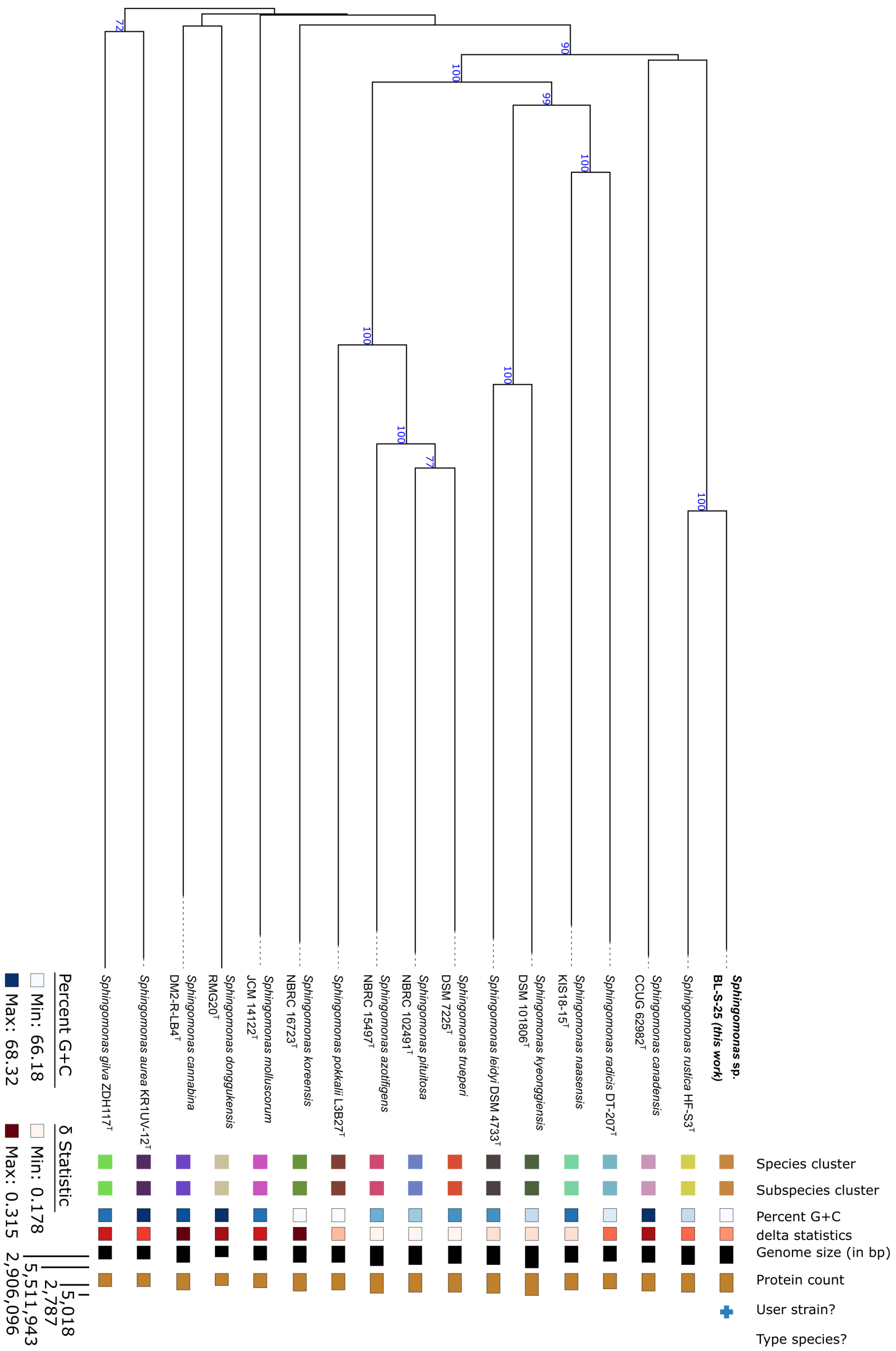

Figure S6. Genome phylogeny tree for *Sphingomonas* sp. BL-S-25. Genome-based tree was made using the TYGS platform, with additional labels appended for clarity. The distance formula was D5 and the distance algorithm was GreedyWithTrimming. Tree inferred with FastME 2.1.6.1 [12] from GBDP (Genome BLAST Distance Phylogeny) distances calculated from genome sequences. The numbers above branches are GBDP pseudo-bootstrap support values >60% from 100 replications (represented in blue numbers), with an average branch support of 79.1%. The tree was rooted at the midpoint [13].

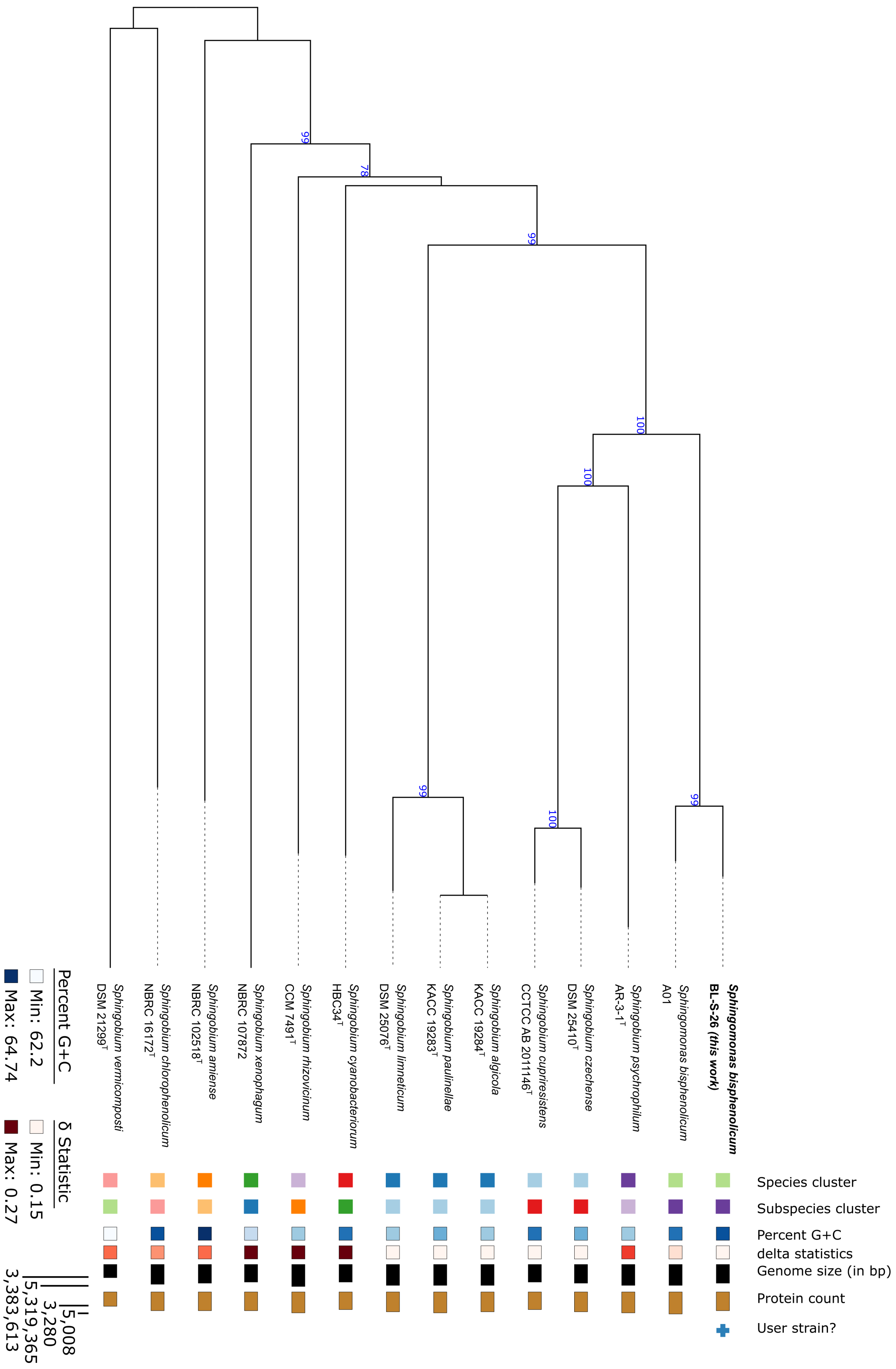

Figure S7. Genome phylogeny tree for *Sphingomonas bisphenolicum* BL-S-26. Genome-based tree was made using the TYGS platform, with additional labels appended for clarity. The distance formula was D5 and the distance algorithm was GreedyWithTrimming. Tree inferred with FastME 2.1.6.1 [12] from GBDP (Genome BLAST Distance Phylogeny) distances calculated from genome sequences. The numbers above branches are GBDP pseudo-bootstrap support values >60% from 100 replications (represented in blue numbers), with an average branch support of 83.4%. The tree was rooted at the midpoint [13].

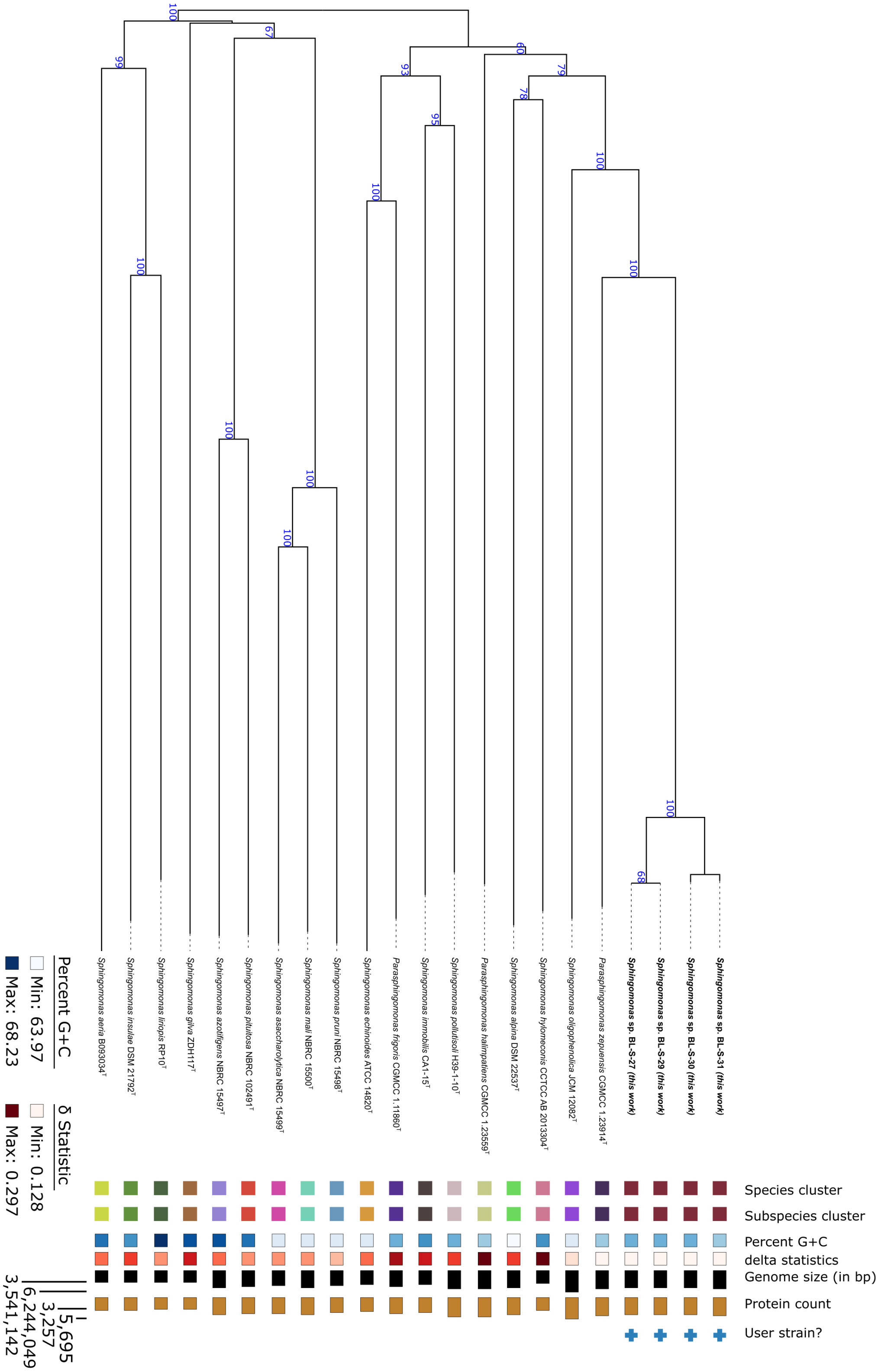

Figure S8. Genome phylogeny tree for *Sphingomonas* sp. BL-S-27, BL-S-29, BL-S-30, and BL-S-31. Genome-based tree was made using the TYGS platform, with additional labels appended for clarity. The distance formula was D5 and the distance algorithm was GreedyWithTrimming. Tree inferred with FastME 2.1.6.1 [12] from GBDP (Genome BLAST Distance Phylogeny) distances calculated from genome sequences. The numbers above branches are GBDP pseudo-bootstrap support values >60% from 100 replications (represented in blue numbers), with an average branch support of 85.2%. The tree was rooted at the midpoint [13].

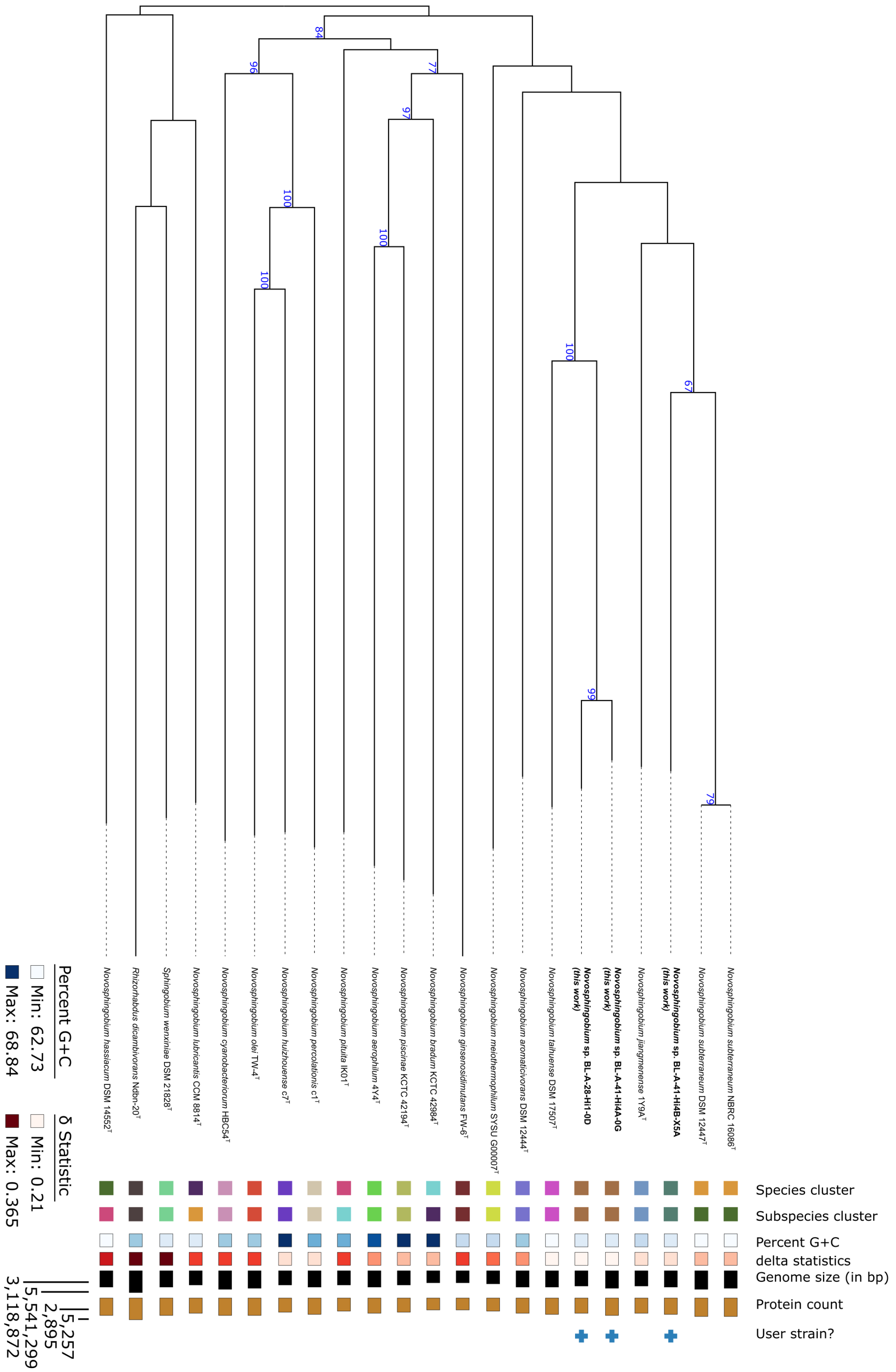

Figure S9. Genome phylogeny tree for *Novosphingobium* spp. strains BL-A-28-Hi1-0D, BL-A-41-Hi4A-0G, and BL-A-41-Hi4B-X5A. Genome-based tree was made using the TYGS platform, with additional labels appended for clarity. The distance formula was D5 and the distance algorithm was GreedyWithTrimming. Tree inferred with FastME 2.1.6.1 [12] from GBDP (Genome BLAST Distance Phylogeny) distances calculated from genome sequences. The numbers above branches are GBDP pseudo-bootstrap support values >60% from 100 replications (represented in blue numbers), with an average branch support of 72.7%. The tree was rooted at the midpoint [13].

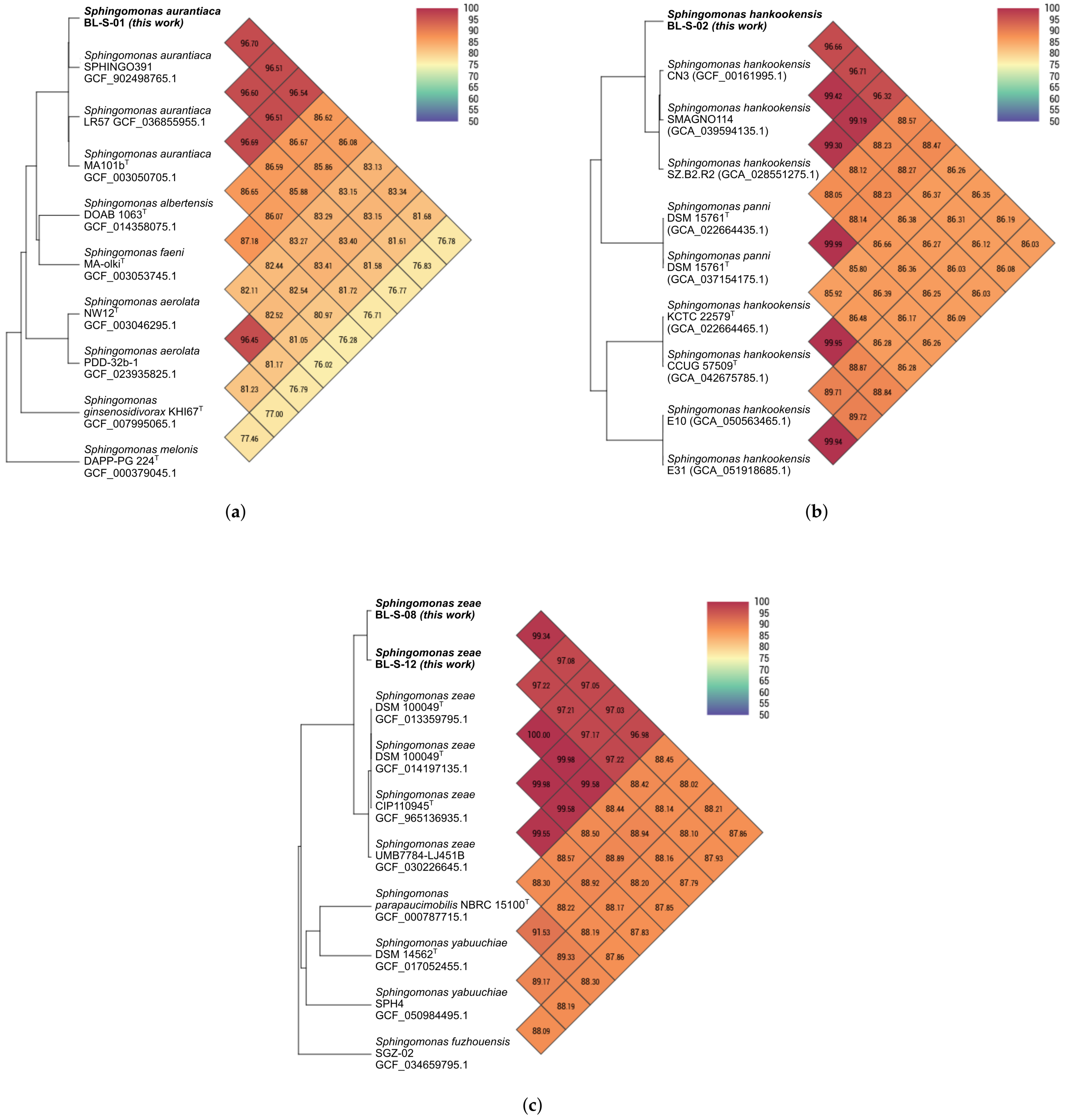

Figure S10. OrthoANI comparison of sequenced genomes assigned as known species against top hits. (**a**) *Sphingomonas aurantiaca* BL-S-01. (**b**) *Sphingomonas hankookensis* BL-S-02. (**b**) *Sphingomonas hankookensis* BL-S-02. (**c**) *Sphingomonas zeae* BL-S-08 and BL-S-12. Heatmaps generated with the OAT [19].

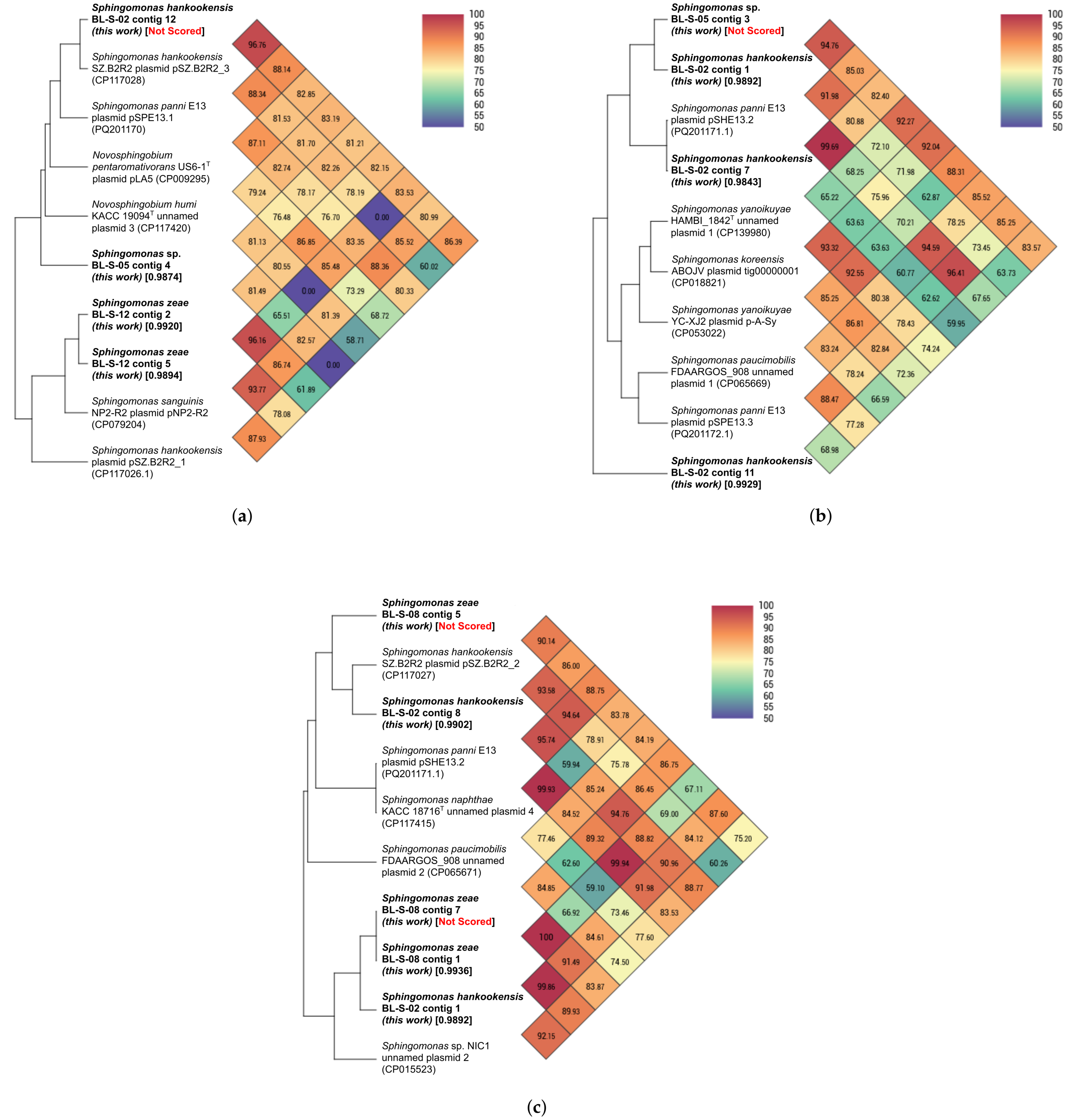

Figure S11. OrthoANI comparison of plasmids unscored by the geNomad analysis. (**a**) *Sphingomonas hankookensis* BL-S-02 contig 12. (**b**) *Sphingomonas* sp. BL-S-05 contig 3. (**c**) *Sphingomonas hankookensis* BL-S-02. (**c**) *Sphingomonas zeae* BL-S-08 contig 5 and contig 7. The geNomad score of plasmids from this work are indicated in brackets [18]. Heatmaps generated with the OAT [19].

References

1. Ahmed, M.M.A.; Tripathi, S.K.; Boudreau, P.D. Comparative metabolomic profiling of *Cupriavidus necator* B-4383 revealed production of cupriachelin siderophores, one with activity against *Cryptococcus neoformans*. *Front. Chem.* **2023**, *11*, 1256962. https://doi.org/10.3389/fchem.2023.1256962.
2. Crumpler, B. Growth Medium Optimization to Promote Siderophore Production and Isolation Using a *Delftia* spp. Model, 2023. Undergraduate Thesis, University of Mississippi, Oxford, MS, USA.
3. ATCC. ATCC Medium 2688: *Acidovorax* Complex Medium (ACM). https://www.atcc.org/~/media/f74db3be206d44de84a32e1dbf9b7108.ashx, n.d. (accessed on 16 October 2025).
4. UTEX Culture Collection of Algae. BG-11 Medium. https://utex.org/products/bg-11-medium?variant=30991786868826, 2009. Recipe improved March 2009. (accessed on 16 October 2025).
5. Meier-Kolthoff, J.P.; Göker, M. TYGS is an automated high-throughput platform for state-of-the-art genome-based taxonomy. *Nature Commun.* **2019**, *10*, 2182. https://doi.org/10.1038/s41467-019-10210-3.
6. Meier-Kolthoff, J.P.; Carbasse, J.S.; Peinado-Olarte, R.L.; Göker, M. TYGS and LPSN: a database tandem for fast and reliable genome-based classification and nomenclature of prokaryotes. *Nucleic Acids Res.* **2022**, *50*, D801–D807. https://doi.org/10.1093/nar/gkab902.
7. Freese, H.M.; Meier-Kolthoff, J.P.; Sardà Carbasse, J.; Afolayan, A.O.; Göker, M. TYGS and LPSN in 2025: a Global Core Biodata Resource for genome-based classification and nomenclature of prokaryotes within DSMZ Digital Diversity. *Nucleic Acids Res.* **2025**, *54*, D884–D891. https://doi.org/10.1093/nar/gkaf1110.
8. Ondov, B.D.; Treangen, T.J.; Melsted, P.; Mallonee, A.B.; Bergman, N.H.; Koren, S.; Phillippy, A.M. Mash: fast genome and metagenome distance estimation using MinHash. *Genome Biol.* **2016**, *17*, 132. https://doi.org/10.1186/s13059-016-0997-x.
9. Lagesen, K.; Hallin, P.; Rødland, E.A.; Stærfeldt, H.H.; Rognes, T.; Ussery, D.W. RNAmmer: consistent and rapid annotation of ribosomal RNA genes. *Nucleic Acids Res.* **2007**, *35*, 3100–3108. https://doi.org/10.1093/nar/gkm160.
10. Camacho, C.; Coulouris, G.; Avagyan, V.; Ma, N.; Papadopoulos, J.; Bealer, K.; Madden, T.L. BLAST+: architecture and applications. *BMC Bioinform.* **2009**, *10*, 421. https://doi.org/10.1186/1471-2105-10-421.
11. Meier-Kolthoff, J.P.; Auch, A.F.; Klenk, H.P.; Göker, M. Genome sequence-based species delimitation with confidence intervals and improved distance functions. *BMC Bioinform.* **2013**, *14*, 60. https://doi.org/10.1186/1471-2105-14-60.
12. Lefort, V.; Desper, R.; Gascuel, O. FastME 2.0: a comprehensive, accurate, and fast distance-based phylogeny inference program. *Mol. Biol. Evol.* **2015**, *32*, 2798–2800. https://doi.org/10.1093/molbev/msv150.
13. Farris, J.S. Estimating phylogenetic trees from distance matrices. *Am. Nat.* **1972**, *106*, 645–668.
14. Kreft, Ł.; Botzki, A.; Coppens, F.; Vandepoele, K.; Van Bel, M. PhyD3: a phylogenetic tree viewer with extended phyloXML support for functional genomics data visualization. *Bioinform.* **2017**, *33*, 2946–2947. https://doi.org/10.1093/bioinformatics/btx324.
15. Meier-Kolthoff, J.P.; Hahnke, R.L.; Petersen, J.; Scheuner, C.; Michael, V.; Fiebig, A.; Rohde, C.; Rohde, M.; Fartmann, B.; Goodwin, L.A.; et al. Complete genome sequence of DSM 30083^T, the type strain (U5/41^T) of *Escherichia coli*, and a proposal for delineating subspecies in microbial taxonomy. *Stand. Genomic Sci.* **2014**, *9*, 2. https://doi.org/10.1186/1944-3277-9-2.
16. Taylor, L. The Design of a PCR-Based Assay to Detect and Isolate the Serine Palmitolytransferase Gene From Environmental Bacteria, 2022. Undergraduate Thesis, University of Mississippi, Oxford, MS, USA.
17. Kolmogorov, M.; Yuan, J.; Lin, Y.; Pevzner, P.A. Assembly of long, error-prone reads using repeat graphs. *Nature Biotechnol.* **2019**, *37*, 540–546. https://doi.org/10.1038/s41587-019-0072-8.
18. Camargo, A.P.; Roux, S.; Schulz, F.; Babinski, M.; Xu, Y.; Hu, B.; Chain, P.S.G.; Nayfach, S.; Kyrpides, N.C. Identification of mobile genetic elements with geNomad. *Nature Biotechnol.* **2024**, *42*, 1303–1312. https://doi.org/10.1038/s41587-023-01953-y.
19. Lee, I.; Kim, Y.O.; Park, S.C.; Chun, J. OrthoANI: An improved algorithm and software for calculating average nucleotide identity. *Int. J. Syst. Evol. Microbiol.* **2016**, *66*, 1100–1103. https://doi.org/10.1099/ijsem.0.000760.

Disclaimer/Publisher's Note: The statements, opinions and data contained in all publications are solely those of the individual author(s) and contributor(s) and not of MDPI and/or the editor(s). MDPI and/or the editor(s) disclaim responsibility for any injury to people or property resulting from any ideas, methods, instructions or products referred to in the content.
